# Comparative Efficacy and Safety of Non-Cryotherapy Treatments Versus Cryotherapy for Plantar Warts: A Systematic Review and Meta-Analysis of Randomized Controlled Trials

**DOI:** 10.3390/jcm15187071

**Published:** 2026-09-11

**Authors:** Aliyah Abdulmohsen Alabdulqader, Suliman Alkhudairy, Abdulelah Saeed Mohammed Alwahabi, Renad Ibrahim Alsulaiman, Hassan A. Alzubaidi, Reem T. Alshammari, Lilian Mazen Abu Dabat, Nawaf Abdullah Alqahtani, Maha Jassim Alhamdan, Ali Bakr, Hesham Alshaikh

**Affiliations:** 1 College of Medicine, Alfaisal University, Riyadh 11533, Saudi Arabia; 2 College of Medicine, Almaarefa University, Riyadh 11597, Saudi Arabia; sulimanalkhudairy9@outlook.com (S.A.); labudabat@gmail.com (L.M.A.D.); 3 College of Medicine, King Saud Bin Abdulaziz University for Health Sciences, Riyadh 14611, Saudi Arabia; abdul4real-@hotmail.com (A.S.M.A.); alqahtani548@ksau-hs.edu.sa (N.A.A.); mahalhamdan55@gmail.com (M.J.A.); 4 College of Medicine, Princess Nourah Bint Abdulrahman University, Riyadh 11671, Saudi Arabia; reenad.alsuliman@gmail.com; 5 Department of Family Medicine, King Abdulaziz Medical City, Jeddah 22384, Saudi Arabia; hassanalzubaidi112@gmail.com; 6 Department of Ophthalmology, Dammam Medical Complex, Dammam 32245, Saudi Arabia; reem.talib98@gmail.com; 7 Faculty of Medicine, Al-Azhar University, Cairo 11754, Egypt; dralibakr93@gmail.com; 8 Department of Dermatology, King Faisal Specialist Hospital & Research Centre, Riyadh 11211, Saudi Arabia; mhalshaikh@kfshrc.edu.sa

**Keywords:** plantar warts, *Verruca plantaris*, *verrucae plantares*, cryotherapy, liquid nitrogen, human papillomavirus

## Abstract

**Background/Objectives**: To systematically evaluate and compare the clinical efficacy and safety of non-cryotherapy modalities versus standard liquid nitrogen cryotherapy for the treatment of plantar warts, while exploring the influence of specific cryotherapy procedural parameters and intervention-related subgroup characteristics on treatment outcomes. **Methods**: A systematic review and meta-analysis of randomized controlled trials (RCTs) was conducted according to PRISMA guidelines. We searched four major databases from inception until 1 July 2026. The primary outcome was complete clearance. Secondary outcomes included partial clearance, recurrence, and treatment-related adverse events. Pooled risk ratios (RR) with 95% confidence intervals (CI) were calculated using random-effects models. **Results**: Thirty-one RCTs involving 2313 patients were included. No statistically significant difference was detected between non-cryotherapy modalities and cryotherapy in complete clearance rates (RR = 1.09, 95% CI: 0.98–1.20; *p* = 0.10; I^2^ = 0.00%) or recurrence outcomes (RR = 0.90, 95% CI: 0.56–1.44; *p* = 0.66). Partial clearance significantly favored cryotherapy (RR = 0.65, 95% CI: 0.44–0.95; *p* = 0.03), particularly in subgroup analyses using two freeze–thaw cycles and treatment intervals of every two weeks. Regarding safety, topical therapies demonstrated significantly lower erythema rates than cryotherapy, while no statistically significant differences were observed in blistering, scarring, or secondary bacterial infection. **Conclusions**: No statistically significant difference was detected between non-cryotherapy modalities and cryotherapy regarding complete clearance or recurrence of plantar warts. Cryotherapy may offer better partial clearance under optimized treatment parameters, whereas topical therapies may be better tolerated in selected patients.

## 1. Introduction

Plantar warts, or verrucae plantares, are benign epidermal tumors resulting from human papillomavirus (HPV) infection, most frequently involving genotypes 1, 2, 4, 27, and 57 [1,2]. They represent a considerable public health burden, with an estimated annual incidence of 14% worldwide [3]. Due to the exceptionally thick *stratum corneum* on the plantar aspect of the foot, these lesions are notoriously recalcitrant and prone to recurrence [4]. Consequently, they frequently cause severe physical pain, significantly restrict weight-bearing daily activities, and drive a high volume of dermatological consultations [5,6].

According to current dermatological guidelines, including those from the British Association of Dermatologists and the Chinese Clinical Guidelines for Cutaneous Warts, liquid nitrogen cryotherapy is consistently listed among the recommended treatment options for cutaneous and plantar warts, alongside other modalities such as salicylic acid, depending on patient age, wart characteristics, tolerability, and clinical context [2,7].

The therapeutic mechanism of cryotherapy relies on inducing localized tissue necrosis and stimulating an inflammatory immune response to eradicate the virus [8]. This physical destruction inevitably damages adjacent healthy perilesional tissue, resulting in significant procedural pain, blistering, and highly variable cure rates that typically average around 45% [9,10].

To overcome these limitations, a broad spectrum of alternative non-cryotherapy modalities has been introduced, ranging from intralesional immunotherapy [11,12] and chemotherapeutic agents [13] to device-based therapies like lasers and microneedling [14,15,16]. The most recent meta-analysis on cryotherapy for plantar warts was limited by substantial clinical and methodological heterogeneity and broad grouping of alternative treatment modalities, resulting in inconclusive findings regarding the relative efficacy of cryotherapy compared with other interventions [4]. Moreover, previous reviews did not comprehensively evaluate the expanding spectrum of contemporary non-cryotherapy therapies alongside detailed cryotherapy-related procedural characteristics, such as application technique, freeze duration, freeze–thaw cycles, and treatment frequency [4,17].

Given the emergence of several recent randomized controlled trials (RCTs) investigating novel topical, intralesional, and device-based therapies, we conducted an updated systematic review and meta-analysis to comprehensively compare the efficacy and safety of non-cryotherapy modalities versus standard cryotherapy for plantar warts, while exploring the potential influence of cryotherapy procedural parameters on treatment outcomes.

## 2. Materials and Methods

### 2.1. Protocol and Registration

We conducted this systematic review and meta-analysis in strict adherence to the Preferred Reporting Items for Systematic Reviews and Meta-Analyses (PRISMA) guidelines and the Cochrane handbook of Systematic Reviews of Interventions [18,19] (Supplementary Table S3).

The protocol was registered in PROSPERO under the registration number **CRD420261419743**.

### 2.2. Eligibility Criteria

We established our inclusion and exclusion criteria based on the PICO framework. We included RCTs that evaluated patients of any age with clinically diagnosed plantar warts, encompassing both treatment-naïve and recalcitrant cases. For the intervention, studies had to administer a non-cryotherapy monotherapy or combination therapy. We accepted topical agents, intralesional agents, device-based therapies, and mechanical/occlusive methods. The comparison group had to receive standard liquid nitrogen cryotherapy applied via spray or cotton swab techniques.

We excluded non-randomized study designs, observational studies, case reports, case series, and narrative or systematic reviews. We also omitted in vitro studies and animal trials. Clinically, we excluded studies targeting non-plantar warts (such as common, flat, or anogenital warts) and papers focusing on unrelated human papillomavirus manifestations, like cervical lesions or oncology. Finally, we excluded articles lacking available full texts. No language restrictions were applied.

### 2.3. Information Sources and Search Strategy

We performed a comprehensive literature search across PubMed, Scopus, Web of Science, and the Cochrane Library from inception to 1 July 2026 using combinations of free-text keywords and MeSH terms. Population-related keywords included “plantar warts,” “*verruca plantaris*,” and “mosaic wart.” Intervention-related terms included “salicylic acid,” “trichloroacetic acid,” “nitric–zinc complex,” “adapalene,” “bleomycin,” “acyclovir,” “imiquimod,” “5-fluorouracil,” “laser,” “Nd:YAG laser,” “CO_2_ laser,” “photodynamic therapy,” “microneedling,” and “duct tape.” Comparator-related terms included “cryotherapy,” “liquid nitrogen,” and “freeze–thaw.” Database-specific syntax adaptations were applied. The full search strategies are provided in Supplementary Table S1.

### 2.4. Study Selection

After retrieving the initial search results, we exported all citations and uploaded them into the Rayyan web application [20]. We utilized Rayyan’s tools to detect and remove duplicate records. Two independent reviewers (A.A., S.A.) then performed a primary screening by evaluating the titles and abstracts of the remaining unique articles to discard visibly irrelevant studies. Following this, the reviewers retrieved the full texts of the potentially relevant papers and assessed them rigorously against our predefined eligibility criteria. When the two reviewers disagreed regarding the inclusion of a specific article, they engaged the senior author to review the paper and reach a final consensus.

### 2.5. Data Extraction

Two reviewers independently (A.S.A., R.I.A.) extracted the data from the selected included RCTs using a standardized spreadsheet. We captured overarching study characteristics, including the first author, publication year, study design, and country of origin. We also extracted baseline demographic and clinical data, such as total sample size, mean age, sex distribution, number of warts, and wart duration. For the treatment outcomes, we recorded the specific non-cryotherapy intervention type, the cryotherapy application technique, the frequency of treatment sessions, the number of freeze–thaw cycles, and the freeze duration per cycle. A third reviewer cross-checked the extracted data against the original publications to eliminate transcription errors and ensure absolute accuracy before analysis.

### 2.6. Risk of Bias Assessment

Because our eligibility criteria restricted inclusion exclusively to RCTs, we evaluated the methodological quality of the included literature using the Cochrane Risk of Bias tool for randomized trials (RoB-2) [21]. Two independent reviewers (H.A.A., R.T.A.) assessed each trial across five domains: the randomization process, deviations from intended interventions, missing outcome data, measurement of the outcome, and selection of the reported result. Based on the signaling questions within the tool, we judged each trial as having a “low risk of bias,” “some concerns,” or a “high risk of bias.” As with previous steps, the reviewers resolved any conflicting judgments through discussion or arbitration by the senior author (H.A.).

### 2.7. Outcome Measures

The primary outcome was complete clearance of plantar warts, assessed at the longest available follow-up. Secondary outcomes included partial clearance and recurrence rates, as well as treatment-related adverse events, including blistering, erythema, scarring, and secondary bacterial infection.

### 2.8. Statistical Analysis

We conducted all quantitative meta-analyses using STATA software, version 19. Since all predefined outcomes were dichotomous, we calculated the risk ratio (RR) alongside its corresponding 95% confidence interval (CI) for each outcome. Anticipating inherent clinical diversity among the different non-cryotherapy modalities and varying cryotherapy application protocols, we strictly applied the restricted maximum likelihood estimation to account for heterogeneity variance for all outcomes [22].

The primary pooled comparison was designed to address the pragmatic clinical question of whether non-cryotherapy alternatives, considered as a broad treatment strategy, differed from standard liquid nitrogen cryotherapy. Given the clinical diversity of the included interventions, this overall estimate was complemented by prespecified subgroup analyses by intervention class and topical mechanism of action and was interpreted as a broad comparative estimate rather than modality-specific evidence.

We measured statistical heterogeneity among the studies using the I-squared (I^2^) statistic and the Cochran Q test. We considered I^2^ value greater than 50% or a Q-test *p*-value below 0.10 as indicative of significant heterogeneity [23].

To explore potential sources of heterogeneity and account for clinical variability, we performed predefined subgroup analyses based on both intervention-related and cryotherapy-related characteristics, as specified in our protocol.

We conducted subgroup analyses based on the type of intervention, including (1) topical therapies; (2) intralesional therapies (bleomycin, acyclovir, vitamin D3, and MMR (Measles, mumps, and rubella) vaccine); (3) device-based therapies (CO_2_ laser, Nd:YAG laser, diode laser, electrosurgery, photodynamic therapy, and laser-induced hyperthermia, as well as drug delivery-assisted techniques such as microneedling-mediated bleomycin or mitomycin); and (4) mechanical or occlusive therapies (duct tape occlusion, dry needling, and related mechanical disruption techniques).

Further subgroup analyses within topical therapies were conducted based on mechanism of action, including (1) keratolytic agents (salicylic acid 40–50%, salicylic acid/lactic acid paint, and salicylic acid ointment formulations); (2) caustic agents (trichloroacetic acid 40%, nitric–zinc complex solution, cantharidin podophyllotoxin-salicylic acid (CPS) formulation, and formaldehyde 10% soaks); (3) retinoids (topical adapalene 0.1% gel or cream); (4) antiviral agents (topical acyclovir cream); (5) immunomodulatory agents (imiquimod 5% cream, alone or in combination with salicylic acid 15%); and (6) chemotherapeutic agents (5-fluorouracil 5% cream), allowing for a detailed mechanistic differentiation between topical treatment modalities.

Additional subgroup analyses were conducted according to detailed cryotherapy-related procedural parameters, including (1) application technique (contact cotton technique, standard spray technique, or device spray technique); (2) frequency (every week, every 2 weeks, every 2–3 weeks, or every 3–4 weeks); (3) number of freeze–thaw cycles per session (single cycle, two cycles, three or more cycles, or variable cycles ranging from 2 to 5 cycles); and (4) freeze duration per cycle (≤10 s, 11–20 s, ≥21 s).

To validate the robustness of our overall pooled estimates, we performed a leave-one-out sensitivity analysis, systematically omitting one study at a time to ensure no single trial skewed the results.

Finally, to detect potential publication bias and small-study effects, we constructed Funnel plots [24]. Furthermore, Galbraith plots were constructed to visually identify specific studies acting as major outliers and contributing disproportionately to the observed inter-study heterogeneity [25]. All reported *p*-values are two-sided, with *p* < 0.05 considered statistically significant unless otherwise specified.

## 3. Results

### 3.1. Literature Search

**Figure 1:**
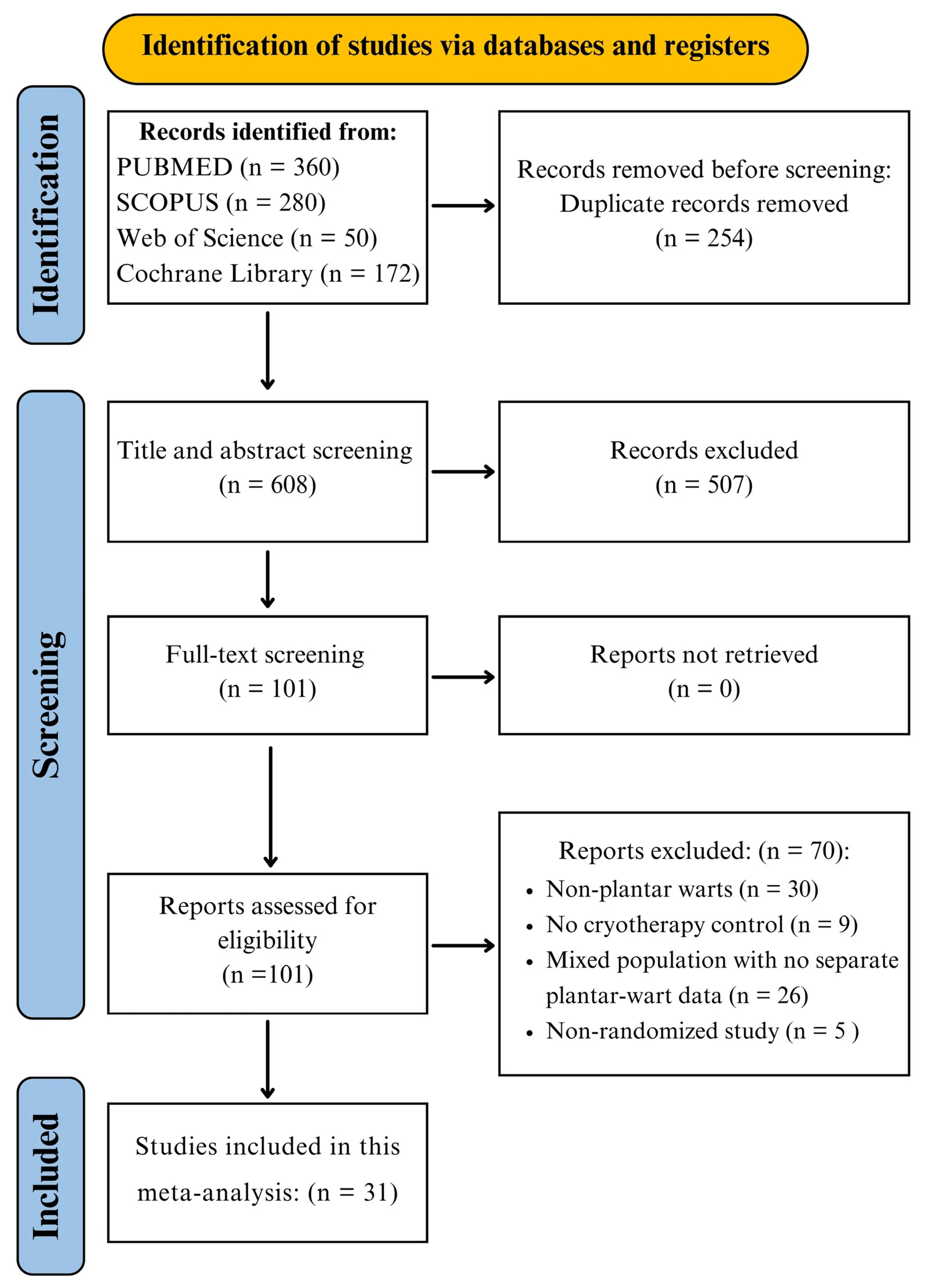
PRISMA flow diagram of study selection. Flow diagram showing study identification, screening, eligibility assessment, and final inclusion of RCTs.

The initial database search yielded a total of 862 records. After removing 254 duplicate records, 608 unique articles underwent title and abstract screening, resulting in the exclusion of 507 visibly irrelevant studies. We retrieved the full texts of the remaining 101 articles for rigorous eligibility assessment. Of these, 70 reports were excluded for several reasons. Finally, 31 RCTs met all inclusion criteria and were included in the meta-analysis. The complete study selection process is illustrated in the PRISMA flow diagram (Figure 1).

### 3.2. Characteristics of Included Studies

The 31 included RCTs [8,10,11,12,13,14,15,16,26,27,28,29,30,31,32,33,34,35,36,37,38,39,40,41,42,43,44,45,46,47,48] enrolled a total of 2313 patients, with 1197 allocated to non-cryotherapy interventions and 1116 to cryotherapy. The trials were conducted across diverse geographical regions, including Europe, Asia, the Middle East, and Australia. Study sample sizes ranged widely from 20 to 240 participants.

The included populations encompassed children, adolescents, and adults, with reported mean or average ages ranging from 8.06 to 51.5 years among studies that reported mean age. Detailed study-level baseline characteristics, including sex distribution, number of warts, and wart duration, are summarized in Supplementary Table S2.

The non-cryotherapy groups evaluated a broad spectrum of modalities: topical therapies, intralesional therapies, device-based therapies, and mechanical/occlusive methods.

In the control groups, all patients received liquid nitrogen cryotherapy. The application techniques varied between standard spray, device spray, and cotton-tipped buds. Session frequencies ranged from weekly to every 4 weeks, utilizing between 1 and 3 freeze–thaw cycles per session with varying freeze durations (ranging from 5 to over 30 s). A comprehensive summary of the study characteristics, baseline demographics, and detailed intervention protocols is provided in Table 1 and Supplementary Table S2.

### 3.3. Risk of Bias Assessment

Using the RoB-2 tool, 5 studies were judged as low overall risk of bias, 13 studies had some concerns, and 13 studies were judged as high risk of bias (Supplementary Figure S1).

### 3.4. Primary Outcome

A total of 29 studies enrolling 2063 patients (1072 in the non-cryotherapy group and 991 in the cryotherapy group) were included in the primary outcome analysis. Two of the 31 included studies—Mahboob et al. [34] and Azzazi et al. [11]—were not included in this specific quantitative synthesis because they did not report dichotomous patient-level complete-clearance data suitable for pooling. Mahboob et al. [34] reported time to clearance as a continuous outcome, whereas Azzazi et al. [11] reported clearance at the lesion level rather than the patient level. Both studies were retained in the systematic review and contributed to other outcome analyses where compatible data were available.

**Figure 2:**
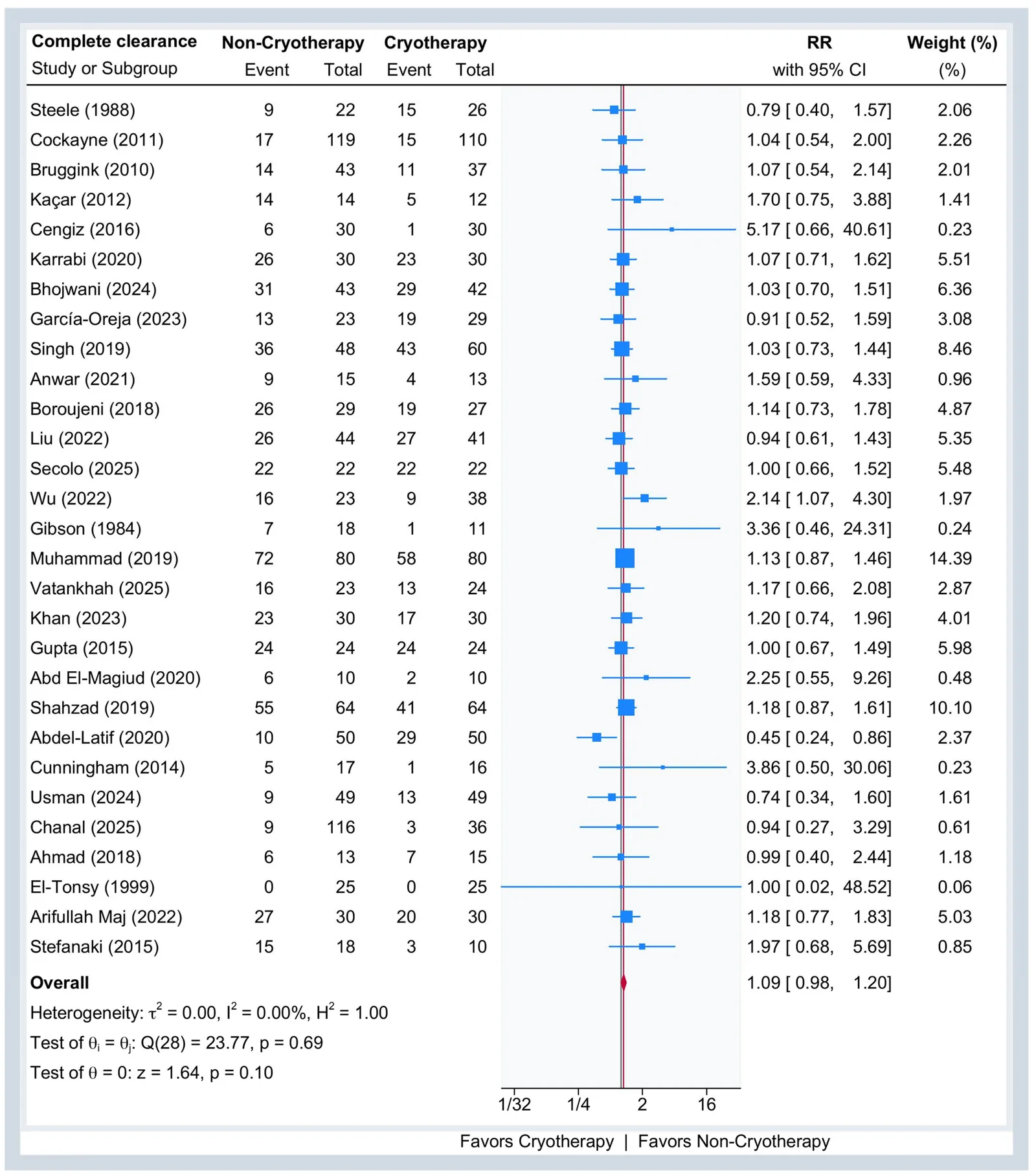
Forest plot of complete clearance. Forest plot comparing complete clearance between non-cryotherapy interventions and cryotherapy using a random-effects model. RR, risk ratio; CI, confidence interval.

The pooled meta-analysis demonstrated no statistically significant difference in complete clearance rates between the two groups (RR = 1.09, 95% CI: 0.98 to 1.20, *p* = 0.10), with negligible heterogeneity across studies (I^2^ = 0.00%, *p* = 0.69) (Figure 2).

**Figure 3:**
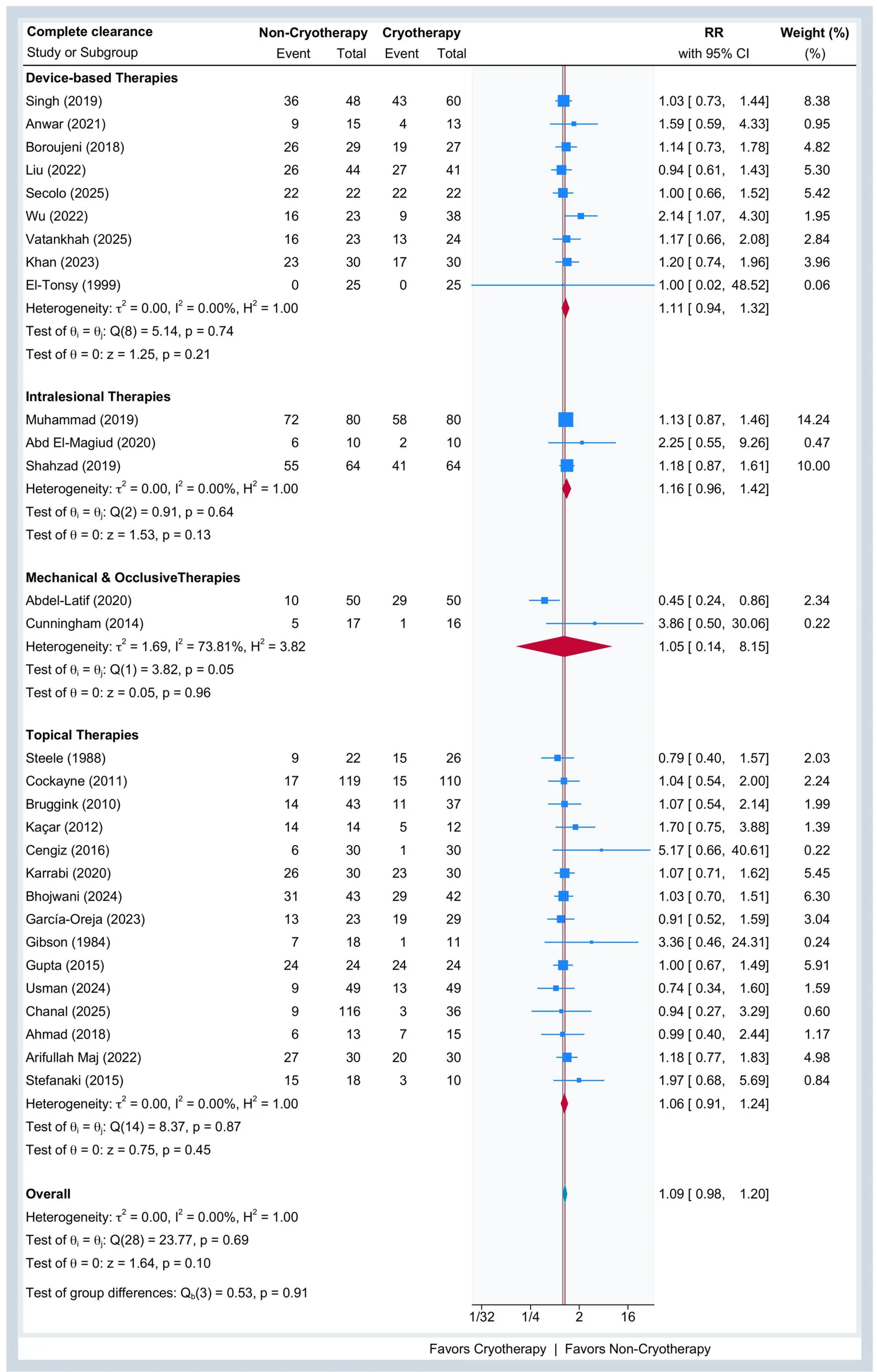
Forest plot of complete clearance by intervention type. Forest plot comparing complete clearance by intervention category using a random-effects model. RR, risk ratio; CI, confidence interval.

Subgroup analysis by intervention type revealed consistent findings, with no statistically significant difference in complete clearance rates identified across any of the four intervention types. Device-based therapies (RR = 1.11, 95% CI: 0.94 to 1.32, *p* = 0.21), intralesional therapies (RR = 1.16, 95% CI: 0.96 to 1.42, *p* = 0.13), mechanical and occlusive therapies (RR = 1.05, 95% CI: 0.14 to 8.15, *p* = 0.96), and topical therapies (RR = 1.06, 95% CI: 0.91 to 1.24, *p* = 0.45) all showed no statistically significant difference, with no statistically significant between-subgroup differences detected (Q_b(3) = 0.53, *p* = 0.91) (Figure 3).

Among topical therapies, further subgroup analysis by mechanism of action demonstrated consistent results across all agent classes, with no statistically significant difference observed for any category. Antiviral agents (single study; RR = 3.36, 95% CI: 0.46 to 24.31, *p* = 0.23), caustic agents (RR = 1.11, 95% CI: 0.83 to 1.48, *p* = 0.48), chemotherapeutic agents (single study; RR = 0.38, 95% CI: 0.04 to 3.51, *p* = 0.40), immunomodulatory agents (RR = 1.32, 95% CI: 0.44 to 4.02, *p* = 0.62), keratolytic agents (RR = 0.98, 95% CI: 0.76 to 1.26, *p* = 0.88), and retinoid agents (RR = 1.08, 95% CI: 0.80 to 1.45, *p* = 0.61) all yielded non-significant estimates, with no statistically significant between-subgroup differences detected (Q_b(5) = 2.75, *p* = 0.74) (Supplementary Figure S2).

Subgroup analysis by cryotherapy application technique similarly demonstrated consistent findings across all techniques, with no statistically significant difference identified for the contact cotton technique (RR = 1.16, 95% CI: 1.00 to 1.35, *p* = 0.06), device spray technique (RR = 0.94, 95% CI: 0.77 to 1.14, *p* = 0.52), or standard spray technique (RR = 1.11, 95% CI: 0.92 to 1.33, *p* = 0.26), and no statistically significant between-subgroup differences detected (Q_b(2) = 2.96, *p* = 0.23) (Supplementary Figure S3).

Subgroup analysis by session frequency showed a statistically significant effect favoring cryotherapy for sessions every 2 weeks (RR = 1.16, 95% CI: 1.01 to 1.32, *p* = 0.03), while no statistically significant difference was observed for sessions every 3–4 weeks (single study; RR = 0.94, 95% CI: 0.61 to 1.43, *p* = 0.76), every 2–3 weeks (RR = 0.81, 95% CI: 0.49 to 1.34, *p* = 0.41), every 4 weeks (single study; RR = 0.94, 95% CI: 0.27 to 3.29, *p* = 0.92), or every week (RR = 1.08, 95% CI: 0.89 to 1.31, *p* = 0.43) (Supplementary Figure S4).

Subgroup analysis by number of freeze–thaw cycles per session demonstrated consistent non-significant findings across all categories, including single cycle (RR = 1.07, 95% CI: 0.93 to 1.23, *p* = 0.34), two cycles (RR = 1.14, 95% CI: 0.94 to 1.37, *p* = 0.18), variable cycles of 2 to 5 (RR = 1.35, 95% CI: 0.60 to 3.02, *p* = 0.47), and three or more cycles (RR = 1.04, 95% CI: 0.77 to 1.40, *p* = 0.82), with no statistically significant between-subgroup differences detected (Q_b(3) = 0.63, *p* = 0.89) (Supplementary Figure S5).

Finally, subgroup analysis by freeze duration per cycle demonstrated consistent non-significant findings for durations of ≤10 s (RR = 1.02, 95% CI: 0.86 to 1.21, *p* = 0.83), 11–20 s (RR = 1.18, 95% CI: 0.89 to 1.56, *p* = 0.25), and ≥21 s (RR = 2.05, 95% CI: 0.54 to 7.73, *p* = 0.29), with no statistically significant between-subgroup differences detected (Q_b(2) = 1.70, *p* = 0.43) (Supplementary Figure S6).

Leave-one-out sensitivity analysis demonstrated that the overall pooled estimate remained generally consistent after sequential omission of individual studies, except for the study by Abdel-Latif et al. [46], the exclusion of which rendered the overall result statistically significant (*p* = 0.041) (Supplementary Figure S7). In addition, a risk-of-bias restriction analysis excluding the 13 studies judged to be at high risk of bias included 16 studies and showed that the primary complete-clearance finding remained statistically non-significant (RR = 1.10, 95% CI: 0.96 to 1.26; *p* = 0.16), with no observed heterogeneity (I^2^ = 0.00%; Q(15) = 10.51, *p* = 0.79) (Supplementary Figure S8). These findings support the robustness of the primary analysis after restriction to studies not judged to be at high risk of bias.

Visual inspection of the funnel plot demonstrated a generally balanced distribution of studies around the pooled estimate, although slight asymmetry was observed, primarily driven by the study by Abdel-Latif et al. [46], with no clear evidence of substantial small-study effects or publication bias for the primary outcome (Supplementary Figure S9).

The Galbraith plot demonstrated that most studies clustered within the 95% confidence bands around the regression line, although the study by Abdel-Latif et al. [46], appeared relatively distant from the central trend, suggesting a potential contribution to the observed variability without representing an extreme outlier (Supplementary Figure S10).

### 3.5. Secondary Outcomes

#### 3.5.1. Partial Clearance

**Figure 4:**
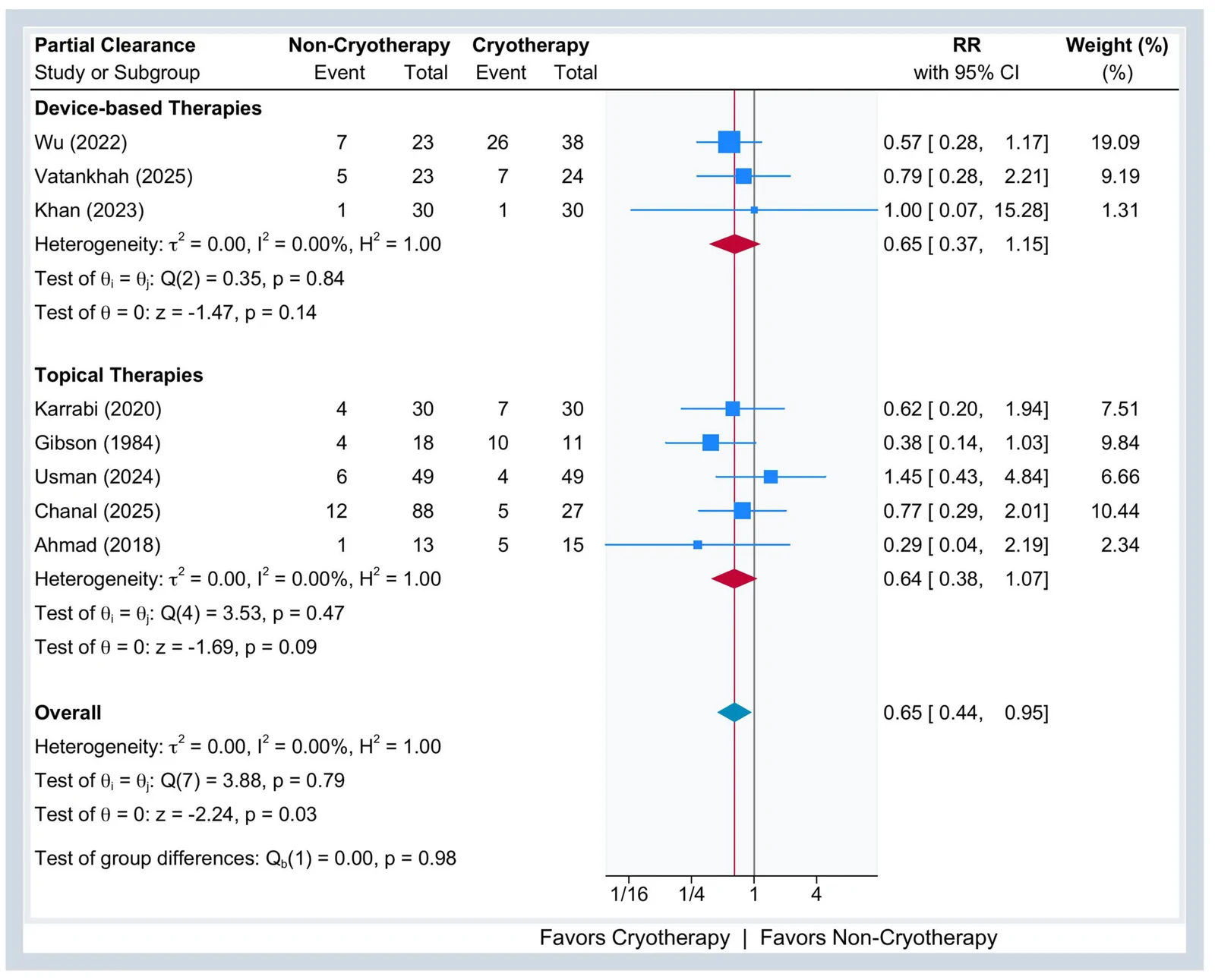
Forest plot of partial clearance by intervention type. Forest plot comparing partial clearance by intervention category using a random-effects model. RR, risk ratio; CI, confidence interval.

The pooled analysis comparing different non-cryotherapy interventions (including device-based and topical therapies) versus cryotherapy for partial clearance demonstrated a statistically significant difference favoring cryotherapy (RR = 0.65, 95% CI: 0.44 to 0.95, *p* = 0.03; I^2^ = 0.00%, *p* = 0.79), although neither device-based therapies (RR = 0.65, 95% CI: 0.37 to 1.15, *p* = 0.14) nor topical therapies (RR = 0.64, 95% CI: 0.38 to 1.07, *p* = 0.09) reached statistical significance individually (Figure 4).

Subgroup analysis by cryotherapy application technique revealed consistent non-significant findings across the contact cotton technique (RR = 0.59, 95% CI: 0.32 to 1.07, *p* = 0.08), device spray technique (RR = 1.00, 95% CI: 0.46 to 2.16, *p* = 0.99), and standard spray technique (RR = 0.54, 95% CI: 0.27 to 1.11, *p* = 0.09) (Supplementary Figure S11).

Subgroup analysis by session frequency identified a statistically significant between-subgroup difference (Q_b(2) = 7.21, *p* = 0.03). Sessions every 2 weeks significantly favored cryotherapy over non-cryotherapy (RR = 0.61, 95% CI: 0.40 to 0.92, *p* = 0.02), while sessions every 2–3 weeks and every 4 weeks, each derived from a single study, showed no statistically significant difference (Supplementary Figure S12).

Subgroup analysis by number of freeze–thaw cycles showed no significant difference with a single cycle and variable cycles (2 to 5 Cycles), while two cycles subgroup significantly favored cryotherapy over non-cryotherapy (RR = 0.59, 95% CI: 0.36 to 0.97, *p* = 0.04) (Supplementary Figure S13).

Subgroup analysis by freeze duration per cycle demonstrated consistent non-significant findings for durations of 11–20 s (RR = 0.55, 95% CI: 0.29 to 1.06, *p* = 0.07), ≤10 s (RR = 0.70, 95% CI: 0.34 to 1.47, *p* = 0.35), and ≥21 s (RR = 0.15, 95% CI: 0.01 to 2.53, *p* = 0.19) (Supplementary Figure S14).

#### 3.5.2. Recurrence Rate

The pooled analysis comparing different non-cryotherapy interventions (including device-based and topical therapies) versus cryotherapy for recurrence rate demonstrated no statistically significant difference between groups (RR = 0.90, 95% CI: 0.56 to 1.44, *p* = 0.66; I^2^ = 0.00%, *p* = 0.90), with similarly non-significant results observed for both device-based therapies (RR = 0.92, 95% CI: 0.52 to 1.61, *p* = 0.76) and topical therapies (RR = 0.86, 95% CI: 0.36 to 2.05, *p* = 0.74) (Supplementary Figure S15).

Among topical therapies, further subgroup analysis by agent class demonstrated consistent non-significant findings for caustic agents (RR = 0.78, 95% CI: 0.18 to 3.35, *p* = 0.74), keratolytic agents (RR = 1.20, 95% CI: 0.20 to 7.30, *p* = 0.84), and retinoid agents (single study; RR = 1.00, 95% CI: 0.02 to 48.45, *p* = 1.00) (Supplementary Figure S16).

Subgroup analysis by cryotherapy application technique demonstrated consistent non-significant findings across the contact cotton technique (RR = 1.06, 95% CI: 0.39 to 2.88, *p* = 0.91), device spray technique (RR = 0.48, 95% CI: 0.16 to 1.49, *p* = 0.21), and standard spray technique (RR = 1.02, 95% CI: 0.55 to 1.86, *p* = 0.96) (Supplementary Figure S17).

Subgroup analysis by session frequency demonstrated consistent non-significant findings for sessions every 2 weeks (RR = 1.06, 95% CI: 0.60 to 1.88, *p* = 0.83), every 2–3 weeks (RR = 0.68, 95% CI: 0.21 to 2.26, *p* = 0.53), and every week (RR = 1.02, 95% CI: 0.09 to 11.50, *p* = 0.98) (Supplementary Figure S18).

Subgroup analysis by number of freeze–thaw cycles demonstrated consistent non-significant findings across single cycle (RR = 0.70, 95% CI: 0.22 to 2.21, *p* = 0.55), two cycles (RR = 0.68, 95% CI: 0.28 to 1.63, *p* = 0.38), variable cycles of 2 to 5 (RR = 0.85, 95% CI: 0.28 to 2.56, *p* = 0.78), and three or more cycles (RR = 1.31, 95% CI: 0.60 to 2.86, *p* = 0.51) (Supplementary Figure S19).

Subgroup analysis by freeze duration per cycle demonstrated consistent non-significant findings for durations of ≤10 s (RR = 1.39, 95% CI: 0.67 to 2.89, *p* = 0.37) and 11–20 s (RR = 0.76, 95% CI: 0.37 to 1.57, *p* = 0.47) (Supplementary Figure S20).

#### 3.5.3. Safety Outcomes (Topical Therapies vs. Cryotherapy)

Topical therapies versus cryotherapy demonstrated consistent safety profiles across all assessed adverse events. No statistically significant difference was observed in blistering (RR = 0.20, 95% CI: 0.03 to 1.41, *p* = 0.11; I^2^ = 68.76%; Supplementary Figure S21), scarring (RR = 0.30, 95% CI: 0.05 to 1.78, *p* = 0.19; I^2^ = 0.00%; Supplementary Figure S22), or secondary bacterial infection (RR = 0.37, 95% CI: 0.05 to 2.69, *p* = 0.32; I^2^ = 0.00%; Supplementary Figure S23). In contrast, erythema was significantly lower in the topical therapy group compared with cryotherapy (RR = 0.15, 95% CI: 0.04 to 0.54, *p* < 0.001; I^2^ = 21.50%; Supplementary Figure S24).

## 4. Discussion

In this systematic review and meta-analysis, we included 31 RCTs comprising 2313 patients with plantar warts, comparing multiple non-cryotherapy modalities with conventional liquid nitrogen cryotherapy. Regarding efficacy outcomes, no statistically significant difference was detected between non-cryotherapy interventions and cryotherapy in complete clearance or recurrence rates, whereas partial clearance significantly favored cryotherapy. In subgroup analyses, cryotherapy application technique, and freeze duration did not show statistically significant differences for complete clearance, partial clearance, or recurrence. Analyses stratified by the number of freeze–thaw cycles also showed no statistically significant subgroup differences for complete clearance or recurrence; however, partial clearance significantly favored cryotherapy in studies using two freeze–thaw cycles per session. Session frequency analyses showed statistically significant findings favoring cryotherapy for complete and partial clearance in studies using two-week intervals, whereas recurrence did not differ significantly across session-frequency subgroups. Similarly, subgroup analyses by intervention type and topical mechanism of action showed no statistically significant subgroup differences for complete clearance or recurrence. Sensitivity analysis demonstrated generally robust findings, although omission of the study by Abdel-Latif et al. [46] rendered the primary outcome statistically significant. Funnel and Galbraith plot assessments showed no major evidence of publication bias, despite slight asymmetry related to Abdel-Latif et al. [46]. Topical therapies showed a more favorable adverse-effect profile with significantly lower erythema rates, while blistering, scarring, and secondary bacterial infection did not differ significantly from cryotherapy.

Our meta-analysis found no statistically significant difference in overall complete clearance or recurrence rates between non-cryotherapy modalities and standard liquid nitrogen cryotherapy. This finding may be explained by the shared therapeutic objective of many available treatments, namely destruction of HPV-infected keratinocytes and stimulation of local immune-mediated clearance [49]. Topically applied keratolytic agents, caustic solutions, and physical devices induce epidermal damage that exposes viral antigens and may stimulate local cell-mediated immune clearance, partly overlapping with the inflammatory response induced by liquid nitrogen cryotherapy [38]. The absence of a statistically significant difference in recurrence between treatment groups may be related to the persistence of latent HPV DNA in the surrounding healthy-appearing skin, which often escapes both focal liquid nitrogen freezing and localized topical applications [50].

The leave-one-out sensitivity analysis showed that exclusion of Abdel-Latif et al. [46] changed the statistical significance of the primary complete-clearance outcome. This influence may be related to the clinically distinct nature of silver duct tape occlusion compared with the more directly tissue-destructive modalities included in the analysis. Silver duct tape occlusion has been proposed to act through occlusion-related maceration and local irritation rather than direct tissue destruction [51]. Therefore, this finding should be interpreted as evidence that the pooled estimate was sensitive to this specific comparison, rather than as a general indication that one treatment category consistently outperformed the other.

Our complete clearance findings partially differ from García-Oreja et al. [4], who reported lower cure rates with cryotherapy than alternative treatments in their overall analysis (OR 0.31, 95% CI: 0.12–0.78), but with substantial heterogeneity (I^2^ = 80%). In subgroup analyses, they reported findings favoring physical treatments and antiviral, chemotherapy, and retinoid therapies over cryotherapy, while keratolytic and placebo/other-treatment comparisons remained inconclusive. They also noted that cryotherapy had lower reported cure rates than several specific non-cryotherapy modalities, including cantharidin-podophyllin-salicylic acid formulation, intralesional bleomycin, laser therapy, topical antivirals, and intralesional immunotherapy. In contrast, our updated meta-analysis found no statistically significant difference in complete clearance between non-cryotherapy modalities and cryotherapy, with negligible heterogeneity. This difference may be related to the larger and more recent evidence base included in our review, differences in included studies and intervention groupings, and the use of RR rather than OR as the summary effect measure. Therefore, our overall pooled estimate should be interpreted as an updated broad comparative estimate and should not be taken to exclude potential advantages of specific non-cryotherapy modalities in individual comparisons.

Partial clearance favored cryotherapy, particularly with two-week intervals and two freeze–thaw cycles per session. This contrasts with García-Oreja et al. [4], who did not identify significant differences between freeze–thaw cryotherapy regimens [37]. The application of two freeze–thaw cycles increases the depth and degree of this cold-induced cellular damage, effectively destroying the thick architecture of plantar warts more rapidly than single cycles or slow-acting topical agents [52]. In addition, the apparent advantage observed with two-week intervals may reflect a more frequent delivery of cryotherapy within the follow-up period, leading to earlier visible wart regression, rather than an independent effect of the interval itself on final cure rates [53].

Topical therapies demonstrated significantly lower erythema rates than cryotherapy. This difference may be explained by the acute thermal injury and inflammatory response induced by liquid nitrogen, compared with the more gradual keratolytic, cytotoxic, or immunomodulatory effects of topical agents.

Regarding the safety outcomes, our analysis demonstrated that topical therapies were associated with a significantly lower incidence of erythema compared to cryotherapy. The lower erythema rates in the topical groups are attributed to the contrasting mechanisms of tissue injury. Cryotherapy induces an acute, severe thermal trauma leading to immediate dermo–epidermal separation, which provokes an intense local inflammatory flare and profound erythema. Conversely, agents like 0.1% adapalene alter keratinization and modulate cell differentiation, while TCA destroys tissue by defolding proteins in a more controlled manner, thereby mitigating acute erythematous reactions and offering a more tolerable recovery for patients [34,54].

The analysis of blistering did not demonstrate a statistically significant between-group difference; however, this outcome showed substantial statistical heterogeneity. This high heterogeneity is attributed to the diverse pharmacological properties of the specific topical agents utilized across the included trials, specifically the difference between caustic agents and keratolytic agents. The trial by Kaçar et al. [36] utilized a caustic proprietary formulation containing CPS; cantharidin is a potent vesicant that naturally degenerates the desmosomal plaque and induces intraepidermal blistering, leading to high blistering rates similar to cryotherapy [36,55,56]. In contrast, the trials evaluating 40% TCA or 50% salicylic acid did not report high blistering rates, as these agents work through gradual keratolysis or protein coagulation rather than immediate vesicle formation, illustrating that the blistering profile depends heavily on the specific chemical agent rather than a class effect of all topical therapies [39,42,57].

Scarring and secondary bacterial infection rates were low in both groups, and neither outcome showed a statistically significant between-group difference, reflecting the superficial tissue effects of standard cryotherapy and controlled topical treatments, which generally spare deeper dermal structures and allow re-epithelialization without permanent architectural damage [58,59].

### 4.1. Strengths and Limitations

This systematic review and meta-analysis have several strengths, including restriction to RCTs, comprehensive assessment of efficacy, adverse events, and subgroup analyses by treatment type and cryotherapy protocol. Compared with García-Oreja et al. [4], our analysis included a larger evidence base (31 RCTs; 2313 patients vs. 14 studies; 1084 patients) and demonstrated negligible heterogeneity in the primary analysis (I^2^ = 0.00% vs. 80%). We also used RR rather than odds ratios, providing a more clinically interpretable estimate for common outcomes such as wart clearance.

Several limitations should be acknowledged. Blinding was limited in many trials because of the physical nature of cryotherapy, raising the possibility of performance and detection bias. In addition, several studies had small sample sizes and relatively short follow-up periods, which limited the assessment of long-term clearance and recurrence. The non-cryotherapy group included clinically diverse interventions with different mechanisms of action, application methods, and treatment intensities; therefore, the overall pooled estimate should be interpreted as a broad comparison rather than evidence for the effect of any single modality. The number of studies within several intervention subgroups was also limited, reducing the precision of modality-specific conclusions. Another important limitation is that the leave-one-out sensitivity analysis showed that exclusion of Abdel-Latif et al. [46] changed the primary complete-clearance result to statistical significance, indicating that the overall inference is sensitive to at least one clinically distinct comparison. Assessment time points, cryotherapy protocols, and adverse-event reporting varied across trials, which may have affected comparability between studies. The included populations also varied by age and wart characteristics. Although age-related differences in immune response may influence treatment outcomes, separate pediatric and adult meta-analyses, or age-stratified subgroup analyses, could not be performed because most trials did not report outcome data separately for children and adults. Similarly, subgroup analyses according to wart chronicity or lesion characteristics were limited by inconsistent reporting across studies. Finally, the lack of routine HPV genotyping precluded analysis of treatment response by HPV subtype.

### 4.2. Clinical Implications

These findings have several clinical implications for plantar wart management. Given the absence of statistically significant differences in complete clearance or recurrence, selected non-cryotherapy modalities may be considered as alternatives, particularly for patients with low pain tolerance or those preferring self-administered home therapies. Topical therapies may offer a less painful option with lower erythema rates. Subgroup analyses suggest that cryotherapy using two freeze–thaw cycles at two-week intervals may improve partial clearance, which may help guide treatment selection for hyperkeratotic or recalcitrant lesions. Clinicians should also counsel patients regarding treatment-specific adverse effects.

Future RCTs should use standardized definitions for complete and partial clearance, include longer follow-up to assess recurrence, and evaluate treatment response according to HPV genotype where feasible. Future trials should also report outcomes separately by age group, wart chronicity, and lesion characteristics to better identify patients most likely to benefit from specific interventions. Importantly, additional head-to-head and add-on trials are needed to determine whether combining standard cryotherapy with adjunctive non-cryotherapy modalities provides incremental benefit over cryotherapy alone in terms of clearance, recurrence, treatment burden, and tolerability.

## 5. Conclusions

This systematic review and meta-analysis found no statistically significant difference between non-cryotherapy modalities and standard liquid nitrogen cryotherapy in complete clearance or recurrence outcomes for plantar warts. Cryotherapy showed favorable partial clearance outcomes in subgroup analyses using two freeze–thaw cycles and two-week treatment intervals. In contrast, topical therapies demonstrated lower erythema rates and may represent a better-tolerated alternative for selected patients. Therefore, treatment selection should remain individualized according to lesion characteristics, patient tolerability, and the risk–benefit profile of each therapeutic modality.

## Figures and Tables

**Table 1:** Summary of included studies.

Study ID	Study Design	Location	Total Sample	Interventions		Cryotherapy				Follow-Up (in Months)	Conclusion
				Intervention Type	Specific Intervention	Cryotherapy Technique	Frequency of Sessions	Aggressiveness (Cycles)	Freezing Duration per Cycle (in Seconds)		
Azzazi (2026) [11]	RCT	Egypt	68	Intralesional	Intralesional Acyclovir	Spray gun	Every 2 weeks	≥3	15	3	Intralesional acyclovir had similar efficacy to cryotherapy but was significantly more painful and required more sessions to reach clearance
Secolo (2025) [30]	RCT	Italy	44	Device-based	Diode Laser	Spray (CryoIQ)	Weekly	1	5	1.25	Diode laser was a faster modality for removing plantar warts compared to cryotherapy. The laser group required significantly fewer sessions. There were no significant differences between the two groups regarding pain or safety/complications
Vatankhah (2025) [16]	RCT	Iran	47	Device-based	Bleomycin via Microneedling	Spray	Every 2 weeks	2	NR	2	Microneedling with topical bleomycin has higher therapeutic efficiency and fewer side effects than cryotherapy for refractory plantar warts. Cryotherapy caused local pain, blisters, and scars, which were absent in the bleomycin group
Chanal (2025) [13]	RCT	France	152	Topical	50% SA ointment, 5% 5-FU cream, 5% Imiquimod cream	Spray (Cry-Ac)	Every 4 weeks (Monthly)	2	5	3	For difficult-to-treat plantar warts, no treatment (50% SA, Cryotherapy, 5-FU, or Imiquimod) showed a substantial positive effect, though SA had the highest remission rate vs. cryotherapy
Bhojwani (2024) [42]	RCT	Pakistan and United Arab Emirates	85	Topical	50% SA	Spray	Every 2–3 weeks	NR	10 to 15	6	50% SA and cryotherapy were both effective, but SA was safer and better tolerated. Cryotherapy achieved results faster but was associated with significantly higher pain scores and recurrence rates. SA is a non-invasive, cost-effective home-based alternative.
Usman (2024) [27]	RCT	Pakistan	98	Topical	50% SA	Spray (Cryogun)	Every 2 weeks	NR	NR	3	There is no significant difference in the efficacy of 50% SA versus cryotherapy for plantar warts
García-Oreja (2023) [10]	RCT	Spain	62	Topical	NZCS	Spray (CryoPen)	Weekly	2	15	3	Cryotherapy and NZCS demonstrated similar efficacy for plantar warts. Cryotherapy was faster, requiring significantly fewer applications to achieve cure, but NZCS was a favorable first-line alternative for those who wish to avoid the pain of cryotherapy
Khan (2023) [45]	RCT	Pakistan	60	Device-based	Mitomycin via Microneedling		Every 2 weeks	1	20	4	Mitomycin microneedling is more effective, requires fewer sessions, and takes less time to complete clearance than cryotherapy. Mitomycin achieved a 76.7% cure rate compared to 56.7% for cryotherapy.
Liu (2022) [15]	RCT	China	113	Device-based	LP Nd:YAG Laser	Spray or Cotton bud	3–4 weeks	2 to 4	NR *	12	LP-Nd:YAG laser and cryotherapy had similar overall cure rates. However, the laser was associated with significantly shorter time to clearance
Wu (2022) [33]	RCT	China	61	Device-based	ALA-PDT	freezing rod	Every 2 weeks	2 to 5	15 to 30	3	5-ALA PDT combined with immunomodulators was superior to cryotherapy for multiple plantar warts. It achieved a significantly higher success rate and a much lower recurrence rate.
Mahboob (2022) [34]	RCT	Pakistan	84	Topical	Topical 0.1% Adapalene	Spray (Cryogun)	Every 2 weeks	2	NR *	NR †	Adapalene 0.1% gel is significantly faster and more effective for complete clearance of plantar warts than cryotherapy
Arifullah Maj (2022) [31]	RCT	Pakistan	60	Topical	Topical 1% Adapalene gel	Dipstick	Every 2 weeks	2	NR	2	Topical 1% Adapalene is more effective and less painful than cryotherapy for complete resolution
Anwar (2021) [43]	RCT	Pakistan	28	Device-based	Electrocautery	Spray	Weekly	2	NR	2	Electrocautery was significantly more effective than cryotherapy for plantar and deep-seated warts. However, cryotherapy caused fewer severe side effects like scarring.
Karrabi (2020) [35]	RCT	Iran	60	Topical	40% TCA	Cotton tip applicator	Every 2 weeks	2	5 to 10	6	40% TCA and cryotherapy had similar effectiveness in resolving plantar warts, though TCA had a numerically higher resolution rate. TCA was considered a superior alternative because it was significantly better tolerated with lower rates of pain, erythema and blistering. Relapse was not observed in either group.
Abd El-Magiud (2020) [47]	RCT	Egypt	20	Intralesional	Intralesional MMR Vaccine	Spray (CRY-AC)	Every 2 weeks	1	30	1	Intralesional MMR vaccine showed a higher complete response rate for plantar warts compared to cryotherapy. Cryotherapy also caused more severe pain and blistering.
Abdel-Latif (2020) [46]	RCT	Egypt	100	Mechanical and Occlusive	Silver Duct Tape	Spray (CryoPro)	Every 2–3 weeks	1	10 to 30	2	Cryotherapy was significantly more effective than silver duct tape occlusion for complete resolution. However, duct tape has fewer side effects
Singh (2020) [29]	RCT	India	147	Device-based	Electrosurgery	Spray	Every 2 weeks	≥3	10	6	Cryotherapy was as effective as electrosurgery for plantar warts but was significantly safer. Electrosurgery resulted in higher levels of pain, delayed wound healing, and scarring. Cryotherapy is considered more suitable for plantar warts due to the lower complication rate
Muhammad (2019) [12]	RCT	Pakistan	160	Intralesional	Intralesional Bleomycin	Cotton swab	Every 2 weeks	1	NR *	1.5	Intralesional bleomycin was significantly more effective than cryotherapy. Cryotherapy was associated with more severe side effects
Shahzad (2019) [32]	RCT	Pakistan	128	Intralesional	Intralesional Vitamin D3	Cotton tip applicator	Weekly	1	10 to 30	1.5	Intralesional Vitamin D3 has significantly higher efficacy compared to cryotherapy
Boroujeni (2018) [14]	RCT	Iran	56	Device-based	CO_2_ Laser	Spray (Cryogun)	Weekly	2	15	3	CO_2_ laser was more efficient and timesaving than cryotherapy. It required significantly fewer sessions for complete clearance. The remission rate was higher for the laser group, although the difference in recurrence at 3 months was not statistically significant
Ahmad (2018) [44]	RCT	Egypt	28	Topical	10% Formaldehyde soaks	Spray (Cry-Ac)	Every 2 weeks	2	15	6	10% Formaldehyde soaks and cryotherapy have almost identical cure rates. Formaldehyde is noted as less costly and painless
Cengiz (2016) [40]	RCT	Turkey	60	Topical	40% TCA	Spray	Every 2 weeks	2	15	1	40% TCA was significantly more effective than cryotherapy for clearing plantar warts. TCA had a much better safety profile, causing significantly less pain, erythema and bullae than cryotherapy.
Gupta (2015) [37]	RCT	India	48	Topical	Topical 0.1% Adapalene	Machine (N2O gas)	Every 2 weeks	1	60 to 120	21	Topical Adapalene (0.1% gel) under occlusion is more effective, safer, and clears plantar warts faster than cryotherapy. Warts cleared in 36.71 days with Adapalene compared to 52.17 days with cryotherapy. Adapalene had zero side effects, whereas cryotherapy caused pain, erythema, infection, and scarring
Stefanaki (2015) [48]	RCT	Greece	28	Topical	5% Imiquimod + 15% SA	Spray gun	Every 2 weeks	2	NR *	3	In children, Imiquimod 5% + SA combination is significantly more effective for plantar warts compared to cryotherapy
Cunningham (2014) [38]	RCT	Australia	37	Mechanical and Occlusive	Dry Needling	Cotton wool swab	Variable (at least 2 weeks apart)	2	5	3	Needling is significantly more effective for the regression of the primary plantar wart compared to cryotherapy. No difference in pain or satisfaction was noted
Kaçar (2012) [36]	RCT	Turkey	26	Topical	CPS Formulation	Spray	Every 2 weeks	1	NR *	2.5	Topical CPS was significantly more effective and required fewer sessions for complete clearance of plantar warts than cryotherapy. While both treatments caused side effects like bullae and pain, CPS achieved a 100% cure rate.
Cockayne (2011) [5]	RCT	UK and Ireland	240	Topical	50% SA	Spray or probe	Every 2–3 weeks	1	2 to 10	6	There was no evidence of a difference in clinical effectiveness between 50% SA and cryotherapy for plantar warts. Both treatments yielded identical clearance rates at 12 weeks. There were no significant differences in the number of remaining warts or time to clearance.
Bruggink (2010) [41]	RCT	Netherlands	83	Topical	40% SA	Cotton wool	Every 2 weeks	1	2 to 10	6.5	There was no clinically relevant difference in effectiveness between cryotherapy and topical SA. Furthermore, cryotherapy did not result in higher patient satisfaction compared to SA.
El-Tonsy (1999) [8]	RCT	Egypt	50	Device-based	Nd:YAG laser hyperthermia	Cotton-tipped applicator	Every 2 weeks	1	30	1.5	Nd:YAG Laser (Hyperthermia) is more effective at eradicating HPV DNA than cryotherapy, though clinical regression takes time for both
Steele (1988) [26]	RCT	UK	48	Topical	SA/LA paint	Cotton wool bud	Weekly	2	10	6	LN was not significantly more effective than SA/LA paint for simple plantar warts. LN caused significantly more pain in the initial treatment phase compared to SA. Recurrence was minimal across all groups.
Gibson (1984) [28]	RCT	UK	32	Topical	Acyclovir Cream	Spray	Every 2 weeks	2	5 to 20	2	LN was an ineffective and unpleasant routine treatment for plantar warts. Only 1 out of 11 patients was cleared using LN. Cryotherapy was found to be significantly more painful and less acceptable to patients than cream-based treatment

**RCT**, Randomized Controlled Trial; **NR**, Not Reported; **SA**, Salicylic Acid; **LA**, Lactic Acid; **SA/LA**, Salicylic Acid/Lactic Acid; **CPS**, Cantharidin—podophyllotoxin—salicylic acid; **TCA**, Trichloroacetic Acid; **NZCS**, Nitric-zinc complex solution; **CO_2_**, Carbon Dioxide; **LP Nd:YAG**, Long-pulsed Neodymium-doped Yttrium Aluminum Garnet; **ALA**, Aminolevulinic acid; **PDT**, Photodynamic Therapy; **ALA-PDT**, Aminolevulinic acid Photodynamic Therapy; **LN**, Liquid Nitrogen (cryotherapy); **MMR**, Measles, Mumps, and Rubella; **5-FU**, 5-Fluorouracil; **N2O**, Nitrous Oxide; **HPV**, Human Papillomavirus. * The freezing duration was until forming a 2 mm halo or appearance of whiteness. † This study reported the follow-up duration as weekly from onset of treatment, the exact follow-up duration was not reported.

## Data Availability

All data generated or analyzed during this study are included in this published article [and its Supplementary Information Files].

## Supplementary Materials

The following supporting information can be downloaded at: https://www.mdpi.com/article/10.3390/jcm15187071/s1, Figure S1: Risk of bias assessment. Risk of bias summary for included RCTs using the Cochrane RoB-2 tool. Figure S2: Complete clearance by topical therapy type. Forest plot of complete clearance stratified by topical therapy mechanism. RR, risk ratio; CI, confidence interval. Figure S3: Complete clearance by cryotherapy application technique. Forest plot of complete clearance stratified by cryotherapy application technique. RR, risk ratio; CI, confidence interval. Figure S4: Complete clearance by session frequency. Forest plot of complete clearance stratified by cryotherapy session frequency. RR, risk ratio; CI, confidence interval. Figure S5: Complete clearance by freeze–thaw cycles. Forest plot of complete clearance stratified by number of freeze–thaw cycles. RR, risk ratio; CI, confidence interval. Figure S6: Complete clearance by freeze duration. Forest plot of complete clearance stratified by freeze duration per cycle. RR, risk ratio; CI, confidence interval. Figure S7: Leave-one-out sensitivity analysis. Leave-one-out analysis evaluating the robustness of the pooled complete-clearance estimate. Figure S8: Complete clearance after exclusion of high-risk-of-bias studies. Forest plot comparing complete clearance between non-cryotherapy interventions and cryotherapy after excluding studies judged to be at high risk of bias. RR, risk ratio; CI, confidence interval. Figure S9: Funnel plot for complete clearance. Funnel plot assessing publication bias and small-study effects for complete clearance. Figure S10: Galbraith plot for complete clearance. Galbraith plot assessing heterogeneity and potential outlier studies for complete clearance. Figure S11: Partial clearance by cryotherapy application technique. Forest plot of partial clearance stratified by cryotherapy application technique. RR, risk ratio; CI, confidence interval. Figure S12: Partial clearance by session frequency. Forest plot of partial clearance stratified by cryotherapy session frequency. RR, risk ratio; CI, confidence interval. Figure S13: Partial clearance by freeze–thaw cycles. Forest plot of partial clearance stratified by number of freeze–thaw cycles. RR, risk ratio; CI, confidence interval. Figure S14: Partial clearance by freeze duration. Forest plot of partial clearance stratified by freeze duration per cycle. RR, risk ratio; CI, confidence interval. Figure S15: Recurrence rate. Forest plot comparing recurrence between non-cryotherapy interventions and cryotherapy. RR, risk ratio; CI, confidence interval. Figure S16: Recurrence by topical therapy mechanism. Forest plot of recurrence stratified by topical therapy mechanism. RR, risk ratio; CI, confidence interval. Figure S17: Recurrence by cryotherapy application technique. Forest plot of recurrence stratified by cryotherapy application technique. RR, risk ratio; CI, confidence interval. Figure S18: Recurrence by session frequency. Forest plot of recurrence stratified by cryotherapy session frequency. RR, risk ratio; CI, confidence interval. Figure S19: Recurrence by freeze–thaw cycles. Forest plot of recurrence stratified by number of freeze–thaw cycles. RR, risk ratio; CI, confidence interval. Figure S20: Recurrence by freeze duration. Forest plot of recurrence stratified by freeze duration per cycle. RR, risk ratio; CI, confidence interval. Figure S21: Blistering. Forest plot comparing blistering between topical therapies and cryotherapy. RR, risk ratio; CI, confidence interval. Figure S22: Scarring. Forest plot comparing scarring between topical therapies and cryotherapy. RR, risk ratio; CI, confidence interval. Figure S23: Secondary bacterial infection. Forest plot comparing secondary bacterial infection between topical therapies and cryotherapy. RR, risk ratio; CI, confidence interval. Figure S24: Erythema. Forest plot comparing erythema between topical therapies and cryotherapy. RR, risk ratio; CI, confidence interval. Table S1. Detailed search strategy for each database. Table S2. Baseline characteristics of included studies. Table S3. PRISMA 2020 checklist.

## Abbreviations

HPV	Human papillomavirus
RCTs	Randomized controlled trials
PRISMA	Preferred Reporting Items for Systematic Reviews and Meta-Analyses
PICO	Population, Intervention, Comparison, Outcome
CO_2_	Carbon dioxide
Nd: YAG	Neodymium-doped yttrium aluminum garnet
MMR	Measles, mumps, and rubella
RoB-2	Risk of Bias 2 tool
STATA	Statistics and Data software
RR	Risk ratio
CI	Confidence interval
I^2^	I-squared heterogeneity statistic
Q test	Cochran Q test
ALA-PDT	Aminolevulinic acid photodynamic therapy
CPS	Cantharidin, podophyllotoxin, and salicylic acid
DNA	Deoxyribonucleic acid
MeSH	Medical Subject Headings
