# Supplementary material for: Comparative Efficacy and Safety of Non-Cryotherapy Treatments Versus Cryotherapy for Plantar Warts: A Systematic Review and Meta-Analysis of Randomized Controlled Trials
Source: J Clin Med. 2026 Sep 11;15(18):7071. doi: 10.3390/jcm15187071 (PMC13607903; doi:10.3390/jcm15187071)
Supplement: Supplementary file 1 [file jcm-15-07071-s001.zip › jcm-4491461-supplementary.pdf]

Supplementary Materials

Supplementary Figure S1. Risk of bias assessment using the RoB-2 tool. 3

Supplementary Figure S2. Forest plot of complete clearance stratified by topical therapy types. 4

Supplementary Figure S3. Forest plot of complete clearance stratified by cryotherapy application technique. 5

Supplementary Figure S4. Forest plot of complete clearance stratified by session frequency. .6

Supplementary Figure S5. Forest plot of complete clearance stratified by number of freeze–thaw cycles. 7

Supplementary Figure S6. Forest plot of complete clearance stratified by freeze duration per cycle. 8

Supplementary Figure S7. Leave-one-out sensitivity analysis for complete clearance. 9

Supplementary Figure S8. Complete clearance after exclusion of high-risk-of-bias studies. .10

Supplementary Figure S9. Funnel plot for complete clearance. 11

Supplementary Figure S10. Galbraith plot for complete clearance. 12

Supplementary Figure S11. Forest plot of partial clearance stratified by cryotherapy application technique. 13

Supplementary Figure S12. Forest plot of partial clearance stratified by session frequency. .14

Supplementary Figure S13. Forest plot of partial clearance stratified by number of freeze–thaw cycles 15

Supplementary Figure S14. Forest plot of partial clearance stratified by freeze duration per cycle. 16

Supplementary Figure S15. Forest plot of recurrence rate. 17

Supplementary Figure S16. Forest plot of recurrence rate stratified by topical therapy mechanism of action. 18

Supplementary Figure S17. Forest plot of recurrence rate stratified by cryotherapy application technique. 19

Supplementary Figure S18. Forest plot of recurrence rate stratified by session frequency. 20

Supplementary Figure S19. Forest plot of recurrence rate stratified by number of freeze–thaw cycles....21

Supplementary Figure S20. Forest plot of recurrence rate stratified by freeze duration per cycle. 22

Supplementary Figure S21. Forest plot of blistering....23

Supplementary Figure S22. Forest plot of scarring. 24

Supplementary Figure S23. Forest plot of secondary bacterial infection....25

Supplementary Figure S24. Forest plot of erythema. 26

Supplementary Table S1. Detailed search strategy for each database. 27

Supplementary Table S2. Baseline characteristics of included studies. 30

Supplementary Figure S1. Risk of bias assessment using the RoB-2 tool.

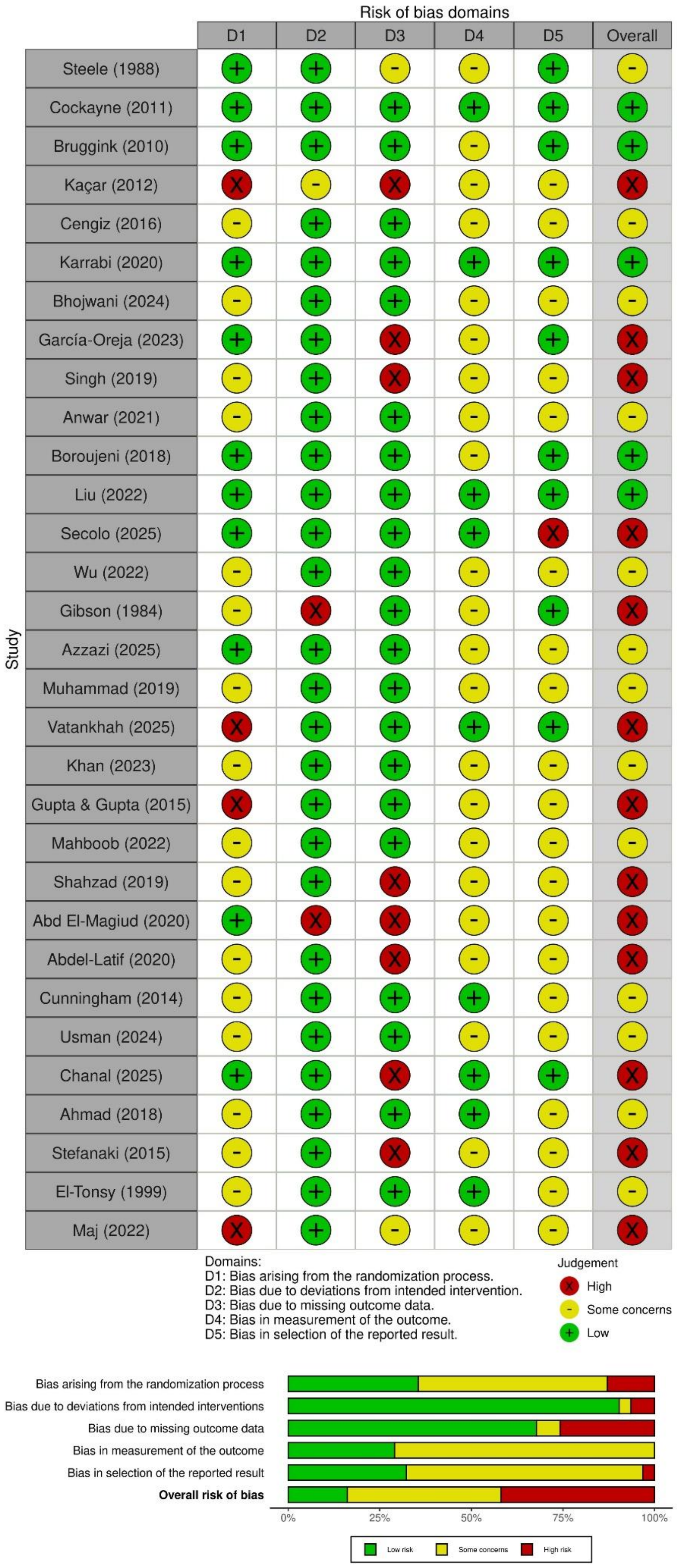

Summary of the methodological quality assessment of the included randomized controlled trials using the Cochrane Risk of Bias tool for randomized trials (RoB-2), across the domains of randomization process, deviations from intended interventions, missing outcome data, measurement of the outcome, selection of the reported result, and overall risk of bias.

RoB-2 = Risk of Bias 2

Supplementary Figure S2. Forest plot of complete clearance stratified by topical therapy types.

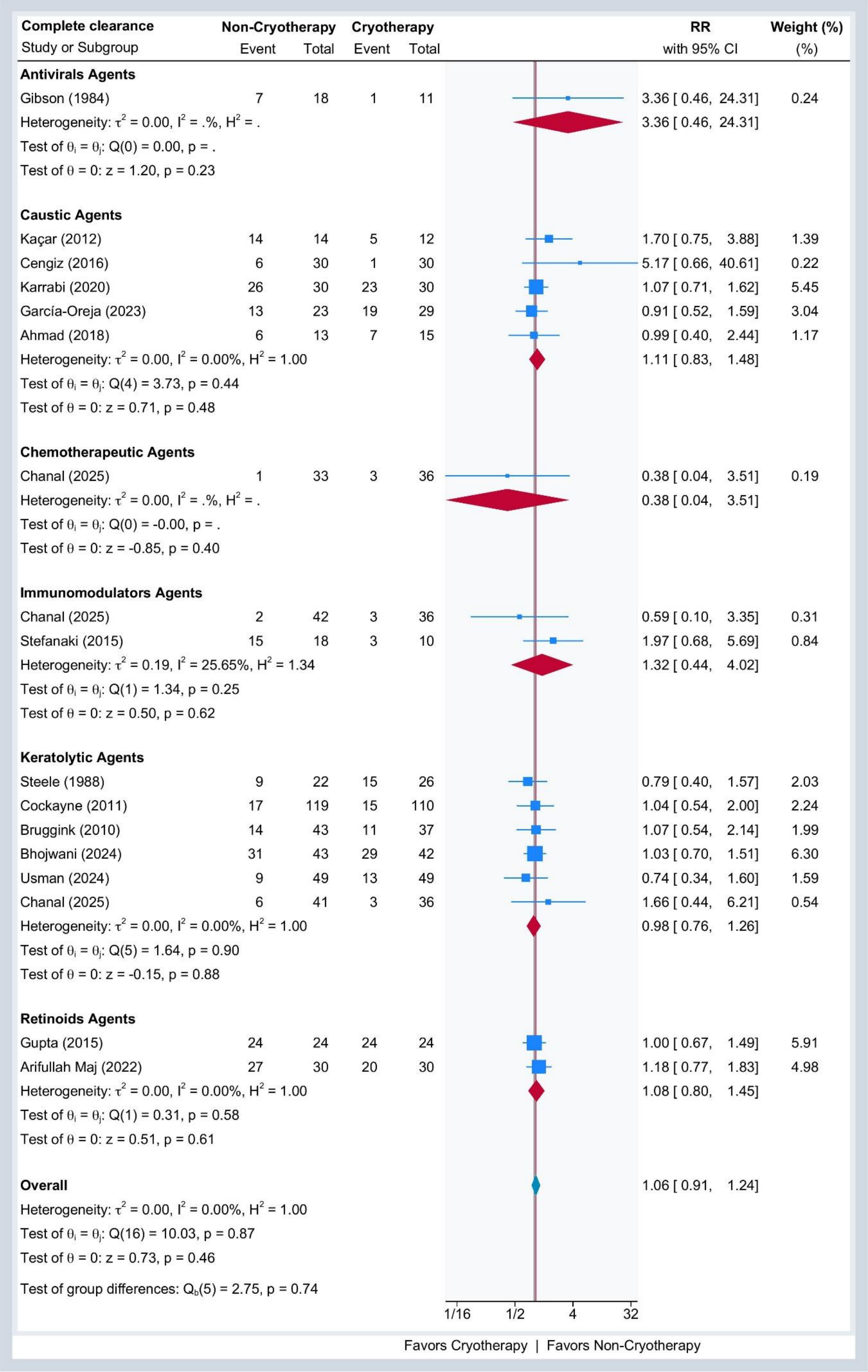

Forest plot comparing complete clearance rates between topical non-cryotherapy interventions and cryotherapy, stratified by mechanism of action (keratolytic, caustic, retinoid, antiviral, immunomodulatory, and chemotherapeutic agents), using a random-effects model.

RR = Risk Ratio; CI = Confidence Interval.

Supplementary Figure S3. Forest plot of complete clearance stratified by cryotherapy application technique.

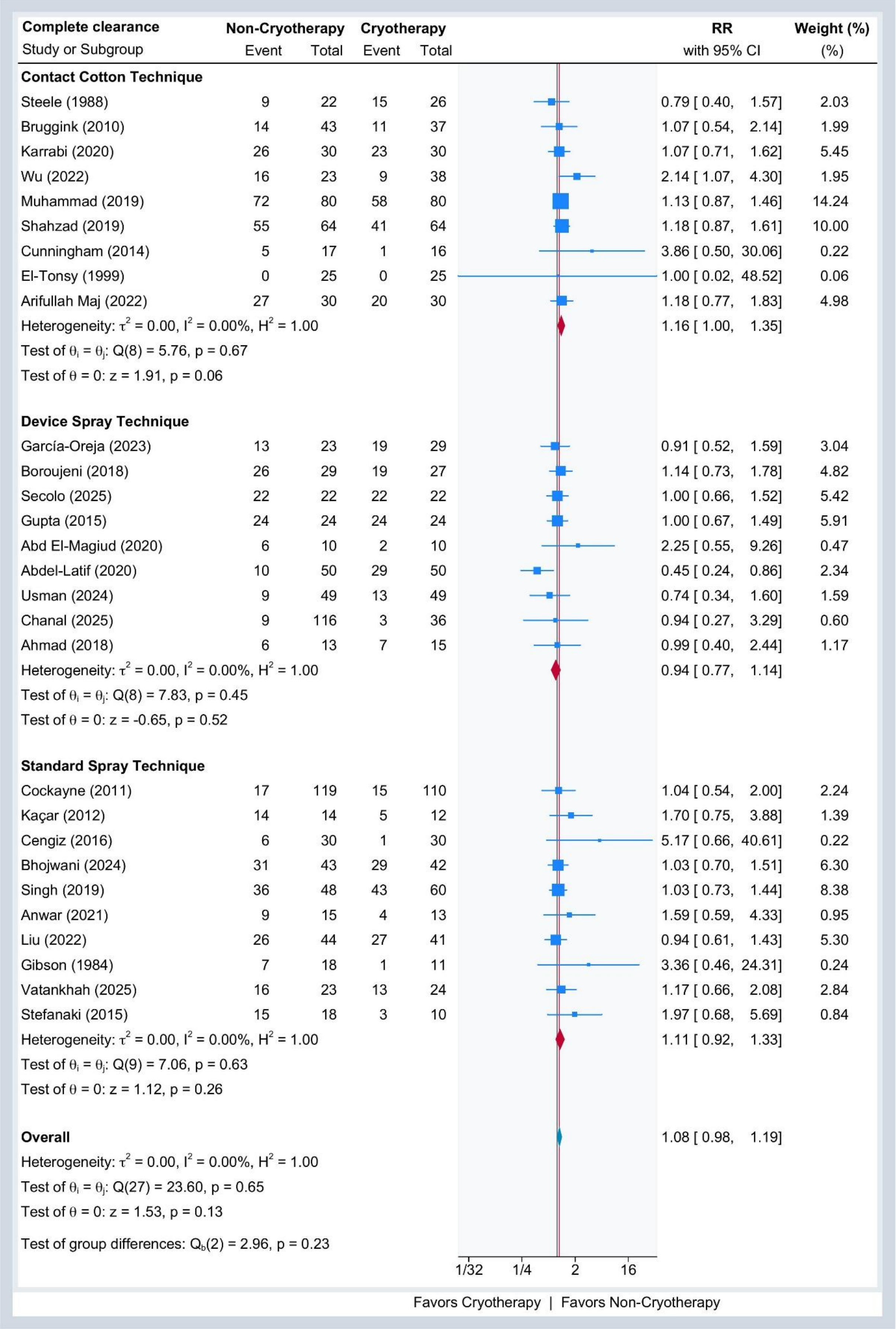

Forest plot comparing complete clearance rates between non-cryotherapy interventions and cryotherapy, stratified by cryotherapy application technique (contact cotton technique, device spray technique, standard spray technique), using a random-effects model.

RR = Risk Ratio; CI = Confidence Interval

Supplementary Figure S4. Forest plot of complete clearance stratified by session frequency.

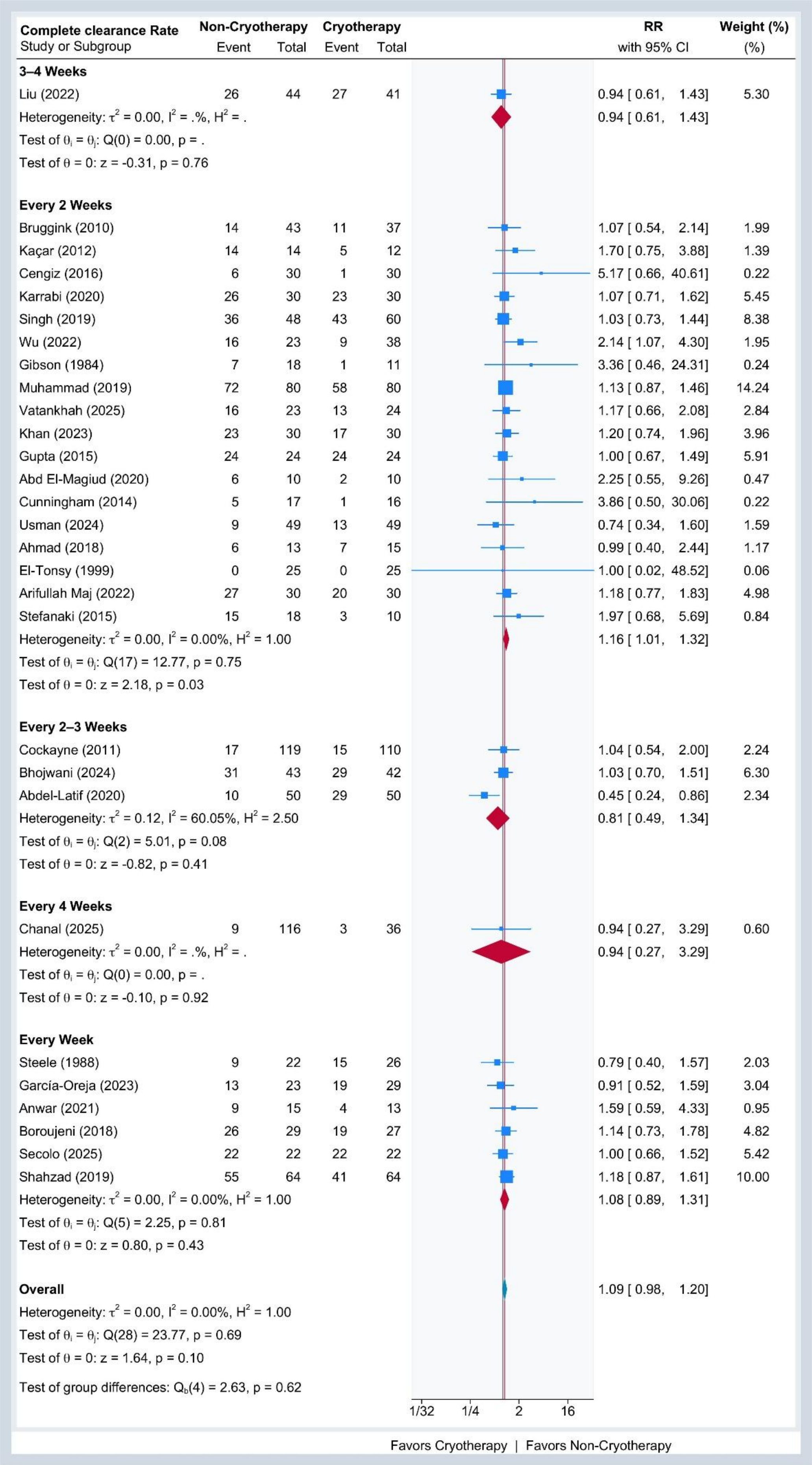

Forest plot comparing complete clearance rates between non-cryotherapy interventions and cryotherapy, stratified according to cryotherapy session frequency, using a random-effects model.

RR = Risk Ratio; CI = Confidence Interval

Supplementary Figure S5. Forest plot of complete clearance stratified by number of freeze–thaw cycles.

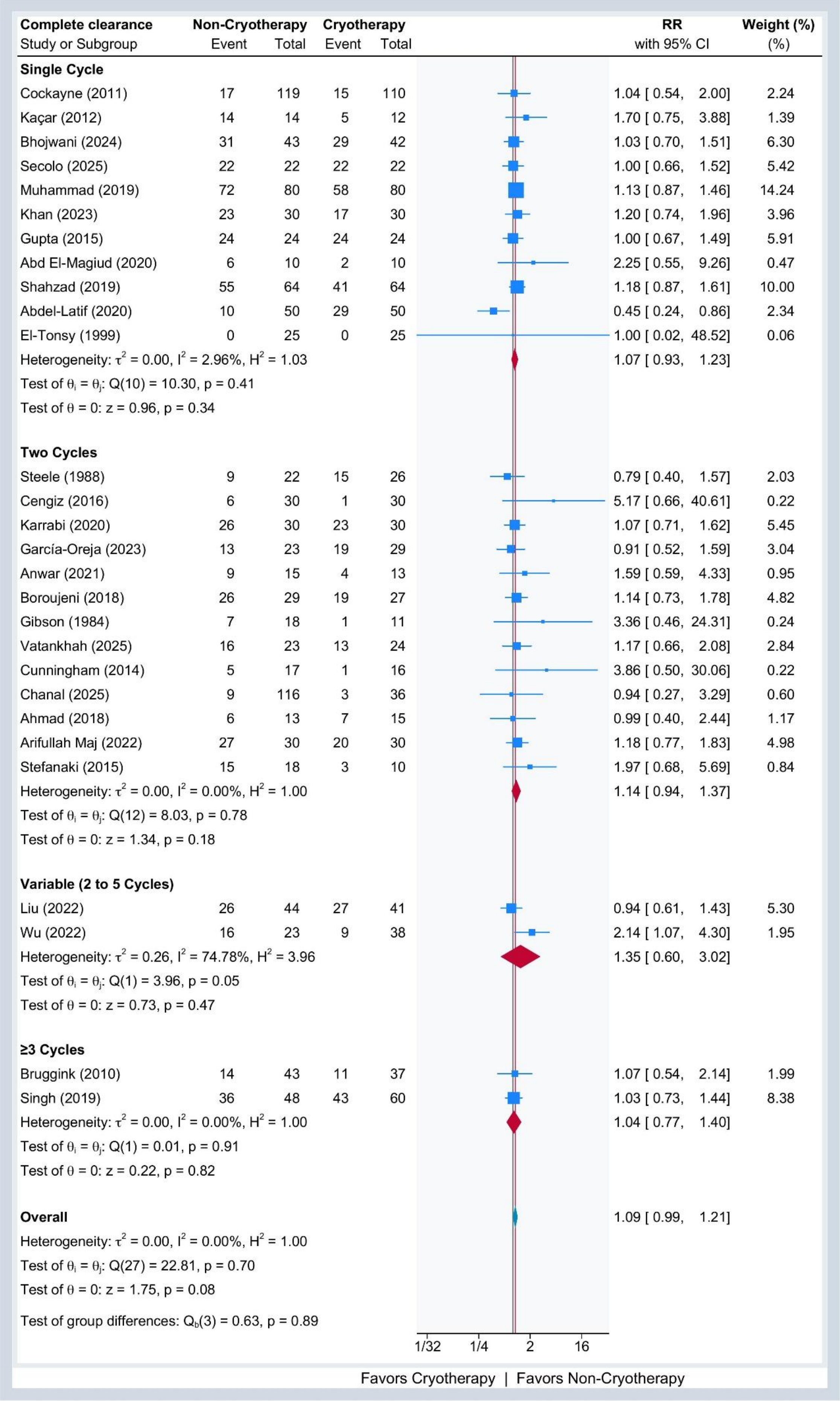

Forest plot comparing complete clearance rates between non-cryotherapy interventions and cryotherapy, stratified according to the number of freeze–thaw cycles per session, using a random-effects model.

RR = Risk Ratio; CI = Confidence Interval

Supplementary Figure S6. Forest plot of complete clearance stratified by freeze duration per cycle.

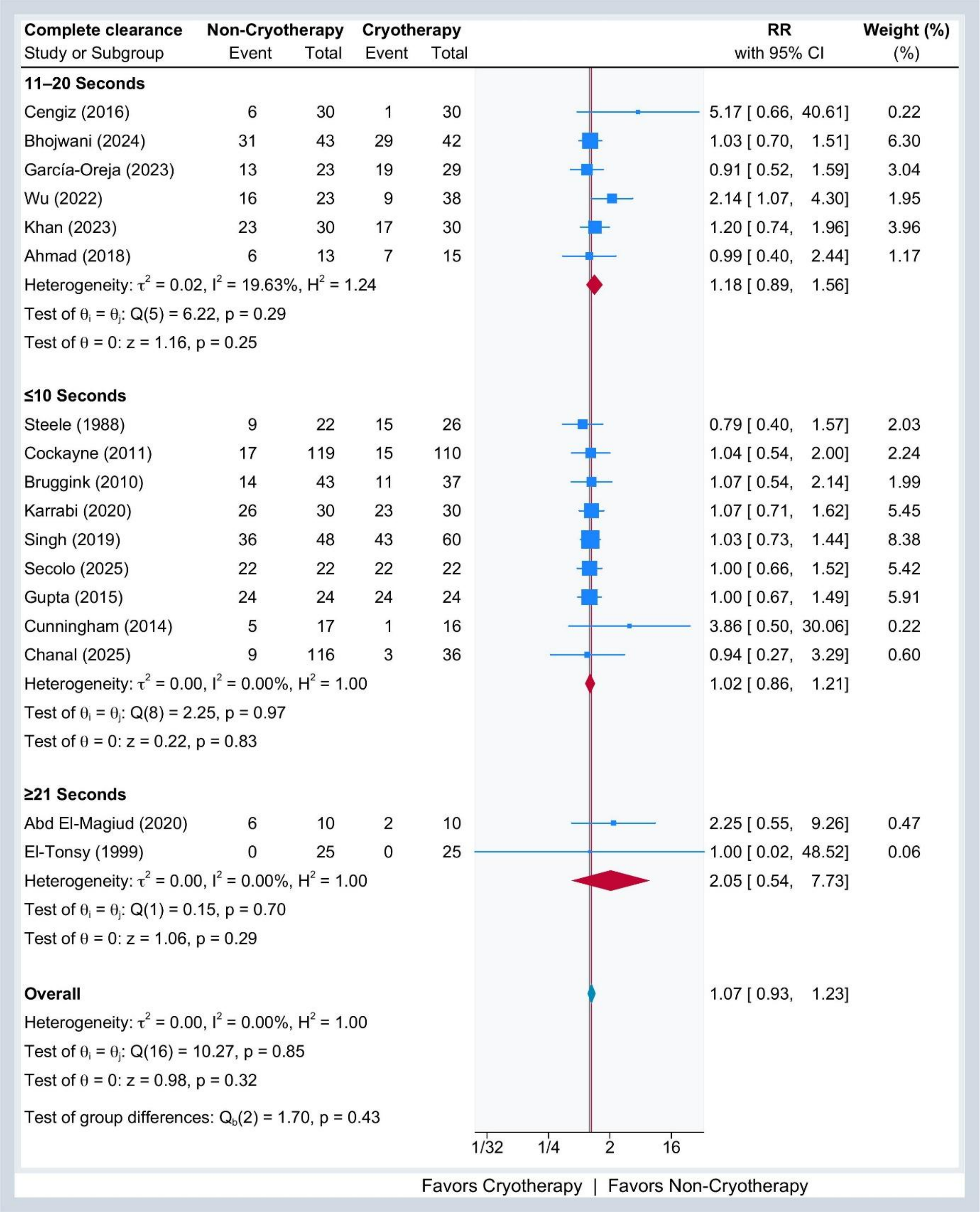

Forest plot comparing complete clearance rates between non-cryotherapy interventions and cryotherapy, stratified according to freeze duration per cycle, using a random-effects model.

RR = Risk Ratio; CI = Confidence Interval

Supplementary Figure S7. Leave-one-out sensitivity analysis for complete clearance.

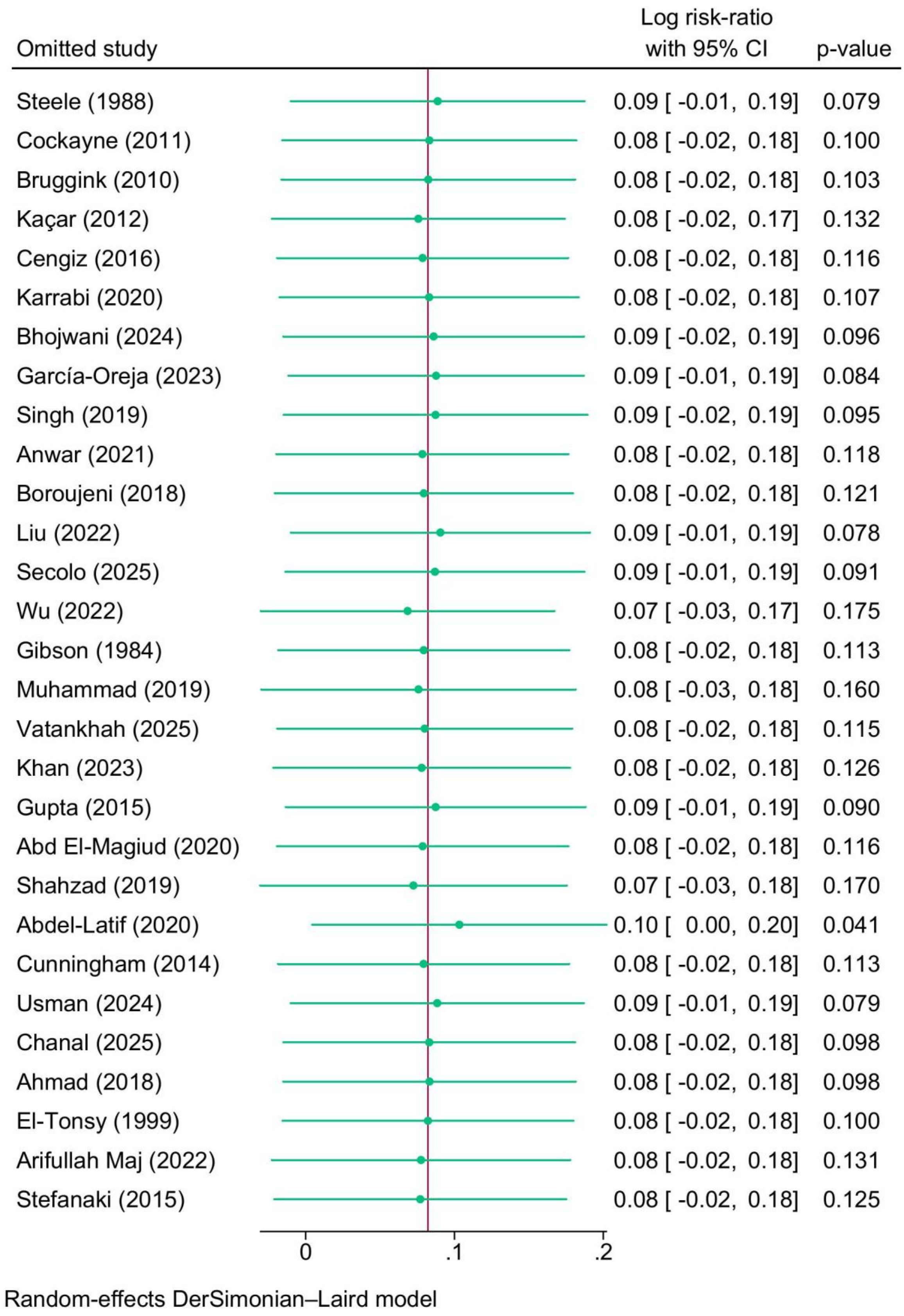

Supplementary Figure S8. Complete clearance after exclusion of high-risk-of-bias studies.

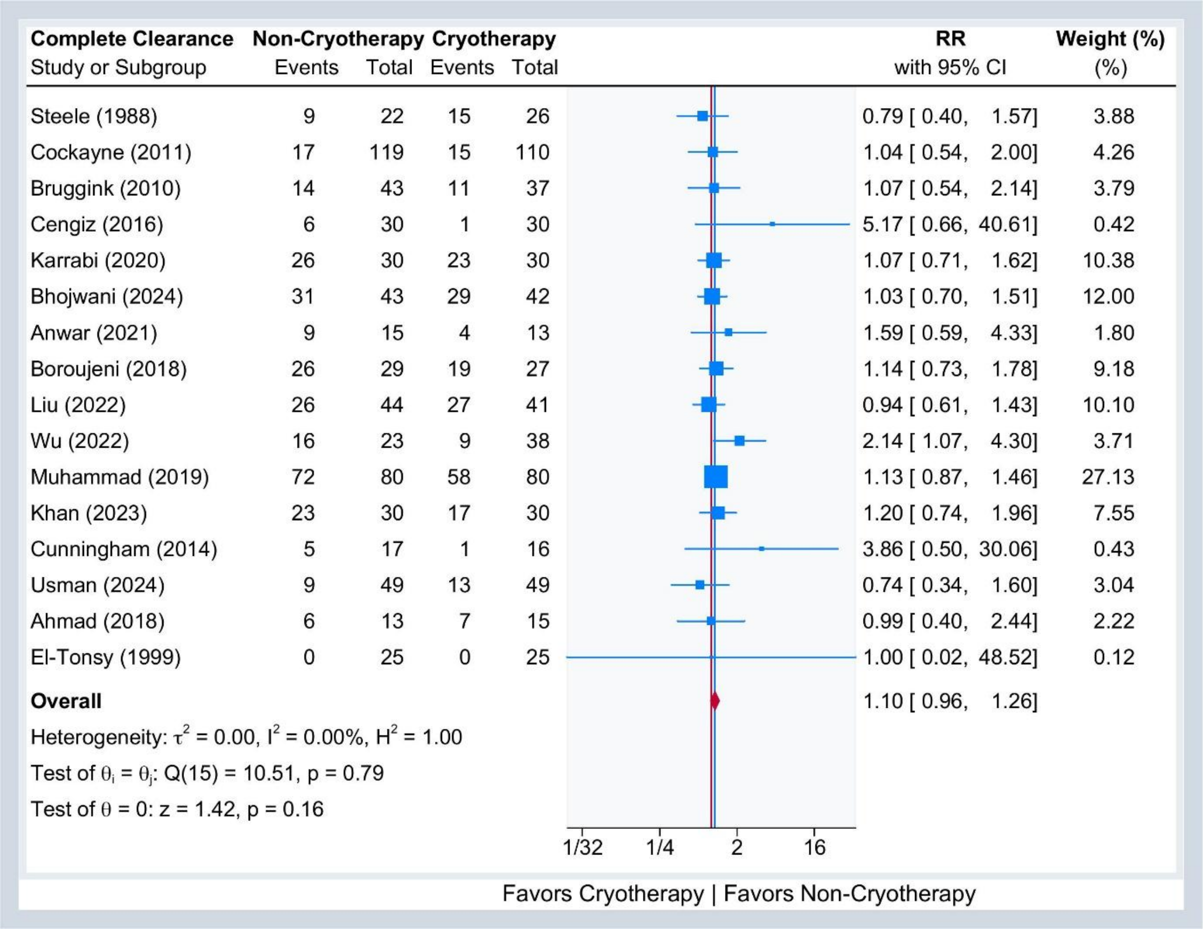

Forest plot comparing complete clearance between non-cryotherapy interventions and cryotherapy after excluding studies judged to be at high risk of bias.

RR, risk ratio; CI, confidence interval.

Supplementary Figure S9. Funnel plot for complete clearance.

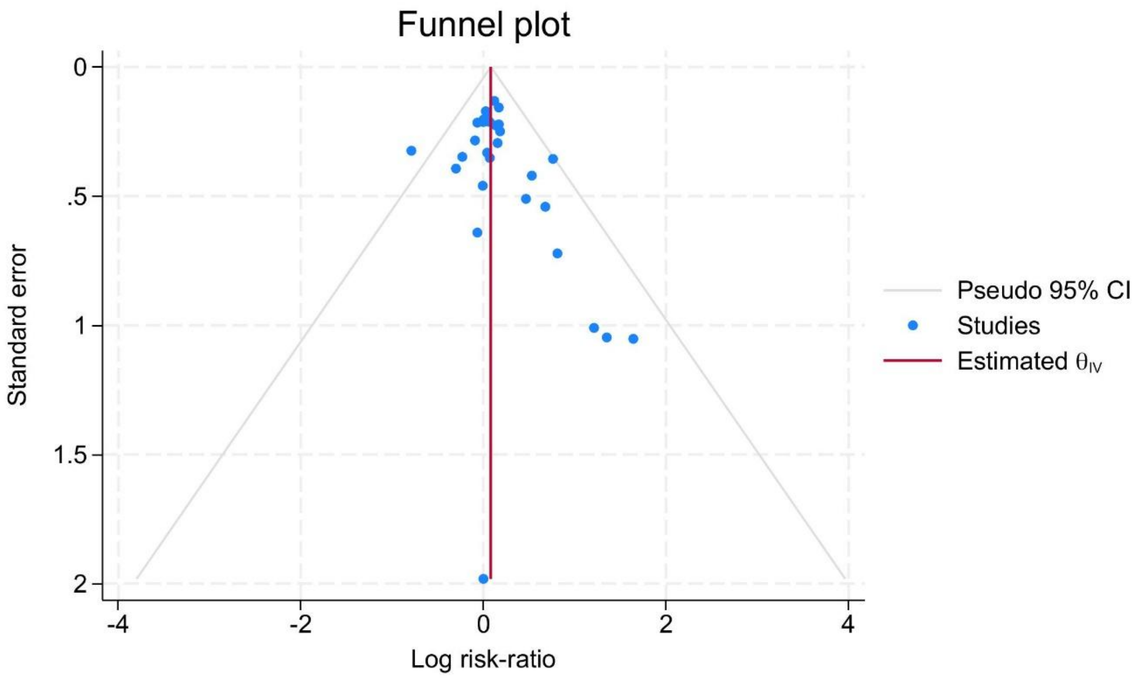

the funnel plot assessing potential publication bias and small-study effects.

Supplementary Figure S10. Galbraith plot for complete clearance.

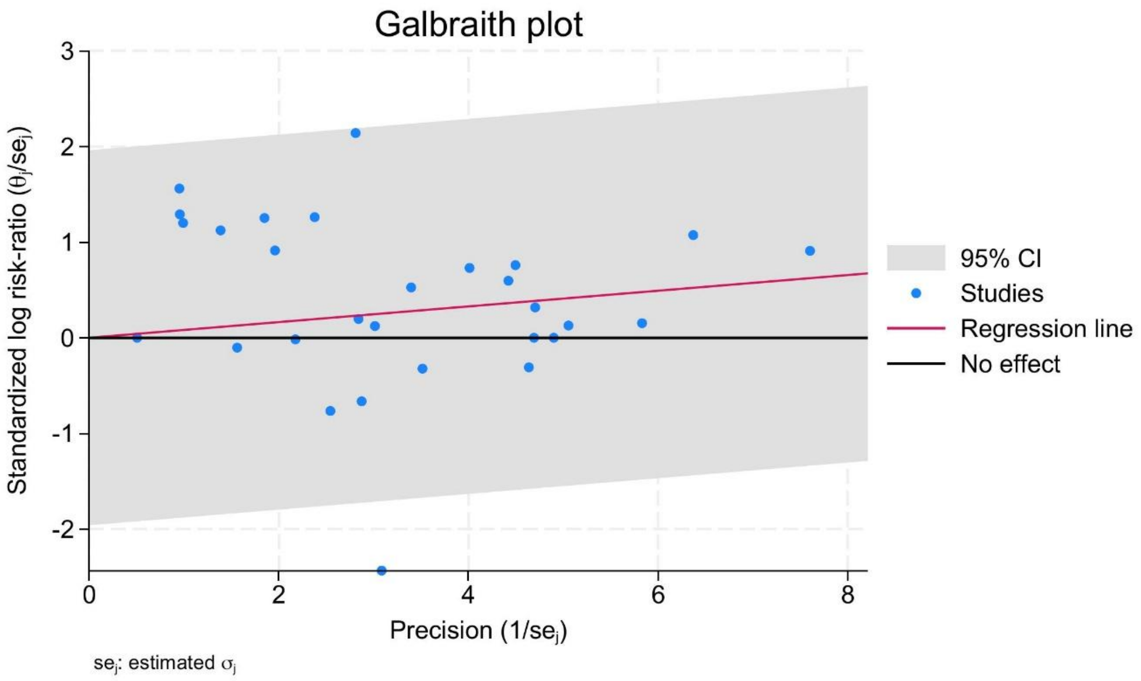

Galbraith plot assessing heterogeneity and identifying potential outlier studies contributing to variability in the pooled analysis of complete clearance.

Supplementary Figure S11. Forest plot of partial clearance stratified by cryotherapy application technique.

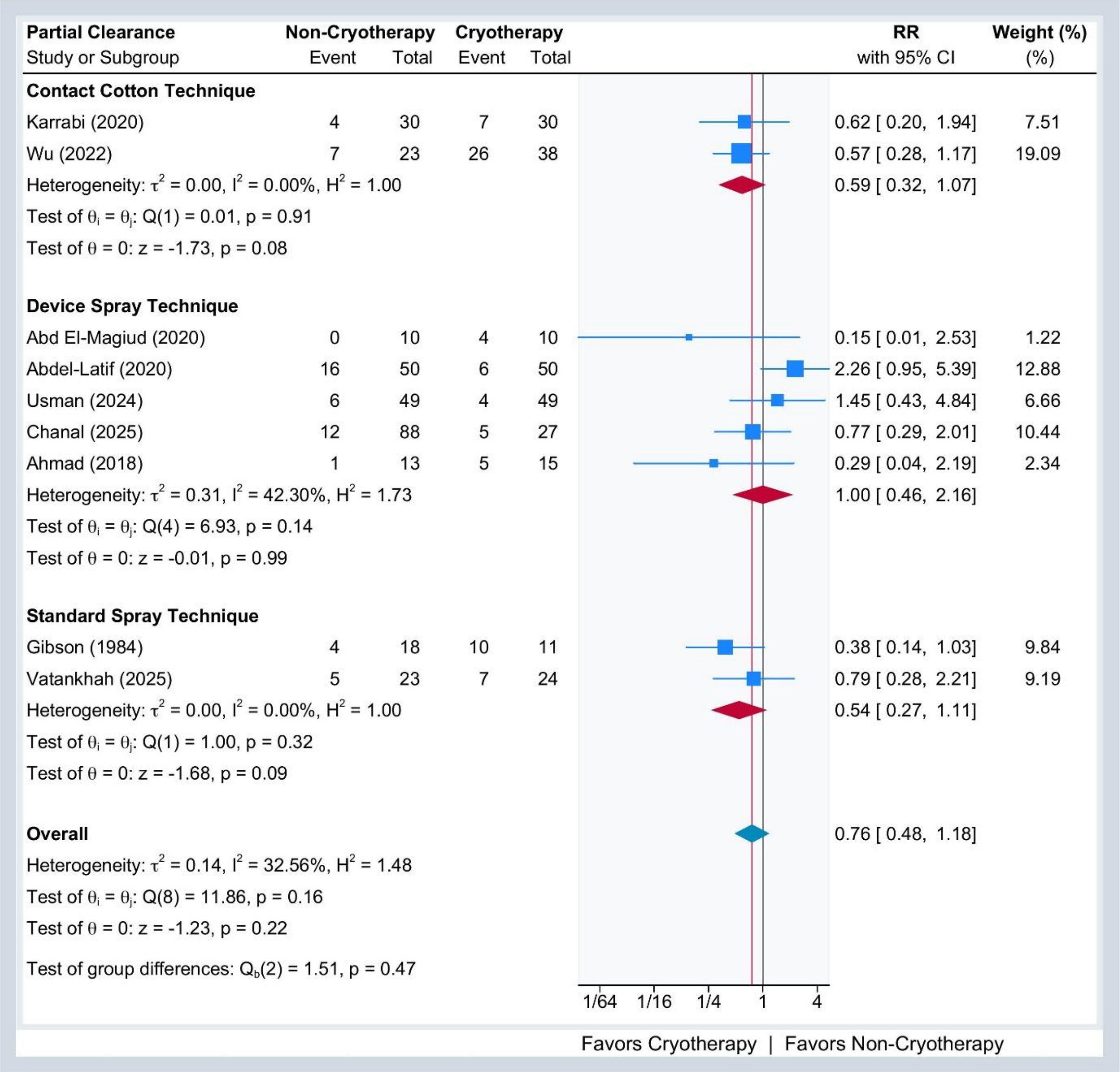

Forest plot comparing partial clearance rates between non-cryotherapy interventions and cryotherapy, stratified by cryotherapy application technique, using a random-effects model.

RR = Risk Ratio; CI = Confidence Interval

Supplementary Figure S12. Forest plot of partial clearance stratified by session frequency.

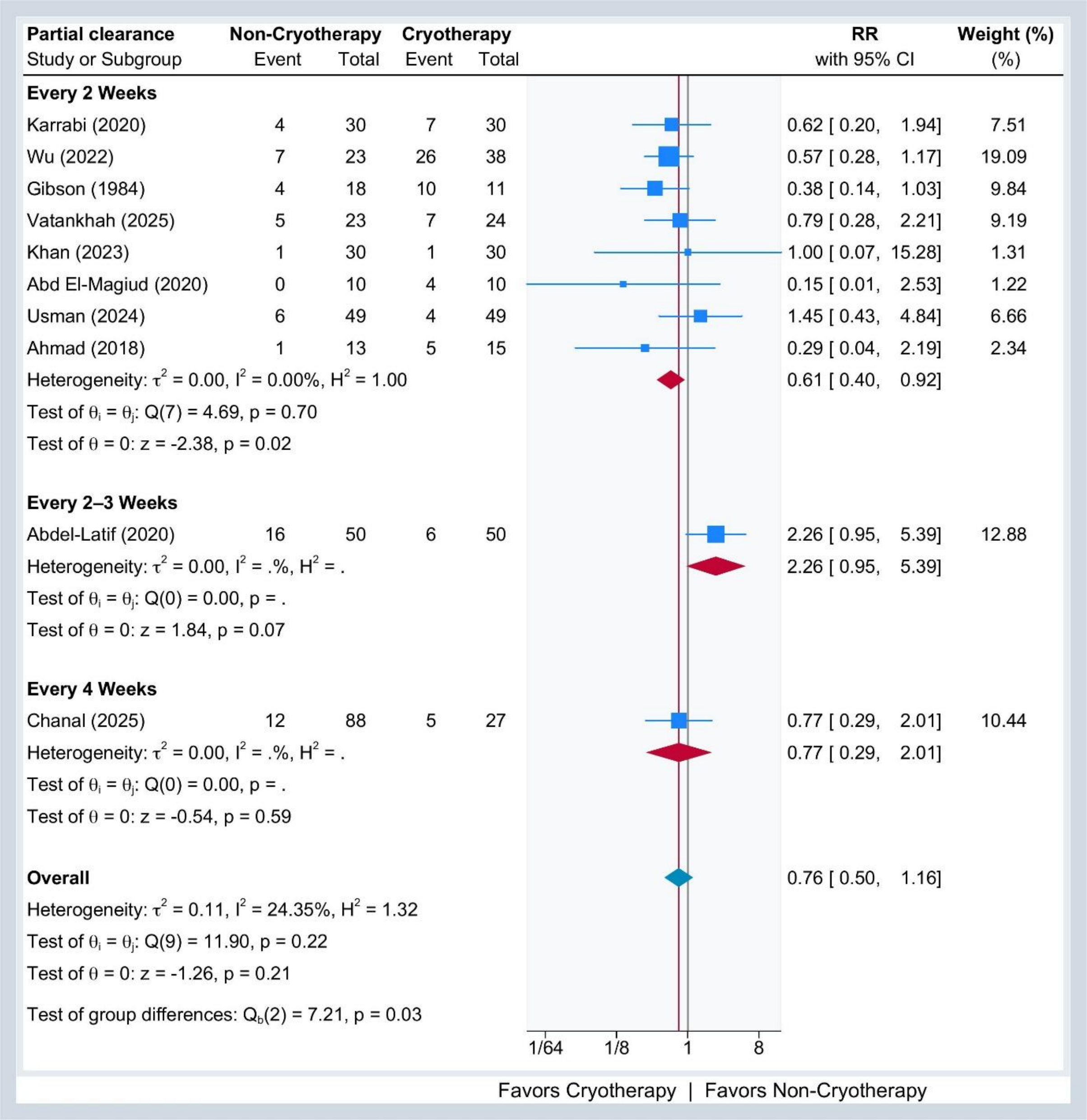

Forest plot comparing partial clearance rates between non-cryotherapy interventions and cryotherapy, stratified according to cryotherapy session frequency, using a random-effects model.

RR = Risk Ratio; CI = Confidence Interval

Supplementary Figure S13. Forest plot of partial clearance stratified by number of freeze–thaw cycles.

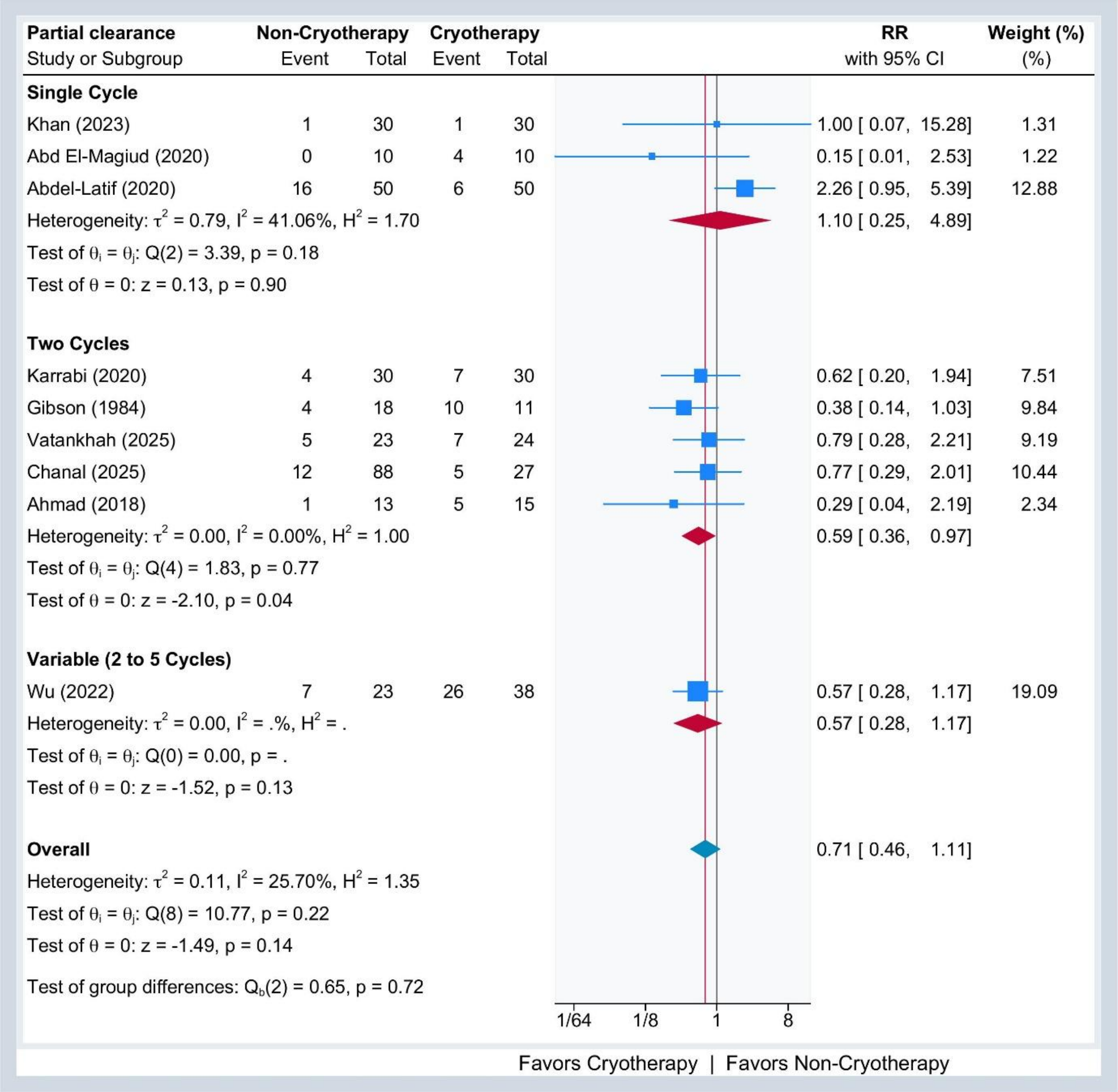

Forest plot comparing partial clearance rates between non-cryotherapy interventions and cryotherapy, stratified according to the number of freeze–thaw cycles per session, using a random-effects model.

RR = Risk Ratio; CI = Confidence Interval

Supplementary Figure S14. Forest plot of partial clearance stratified by freeze duration per cycle.

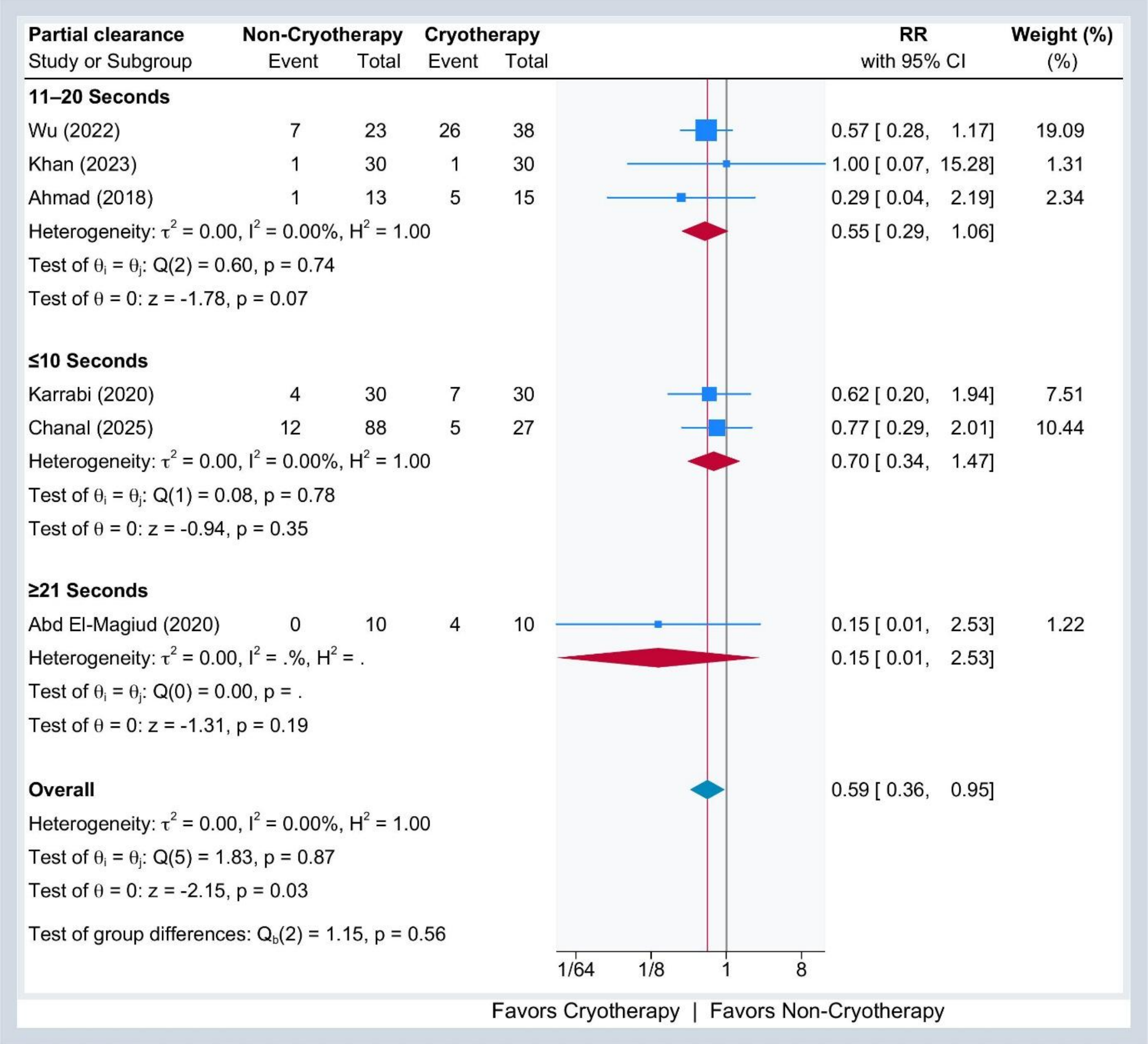

Forest plot comparing partial clearance rates between non-cryotherapy interventions and cryotherapy, stratified according to freeze duration per cycle, using a random-effects model.

RR = Risk Ratio; CI = Confidence Interval

Supplementary Figure S15. Forest plot of recurrence rate.

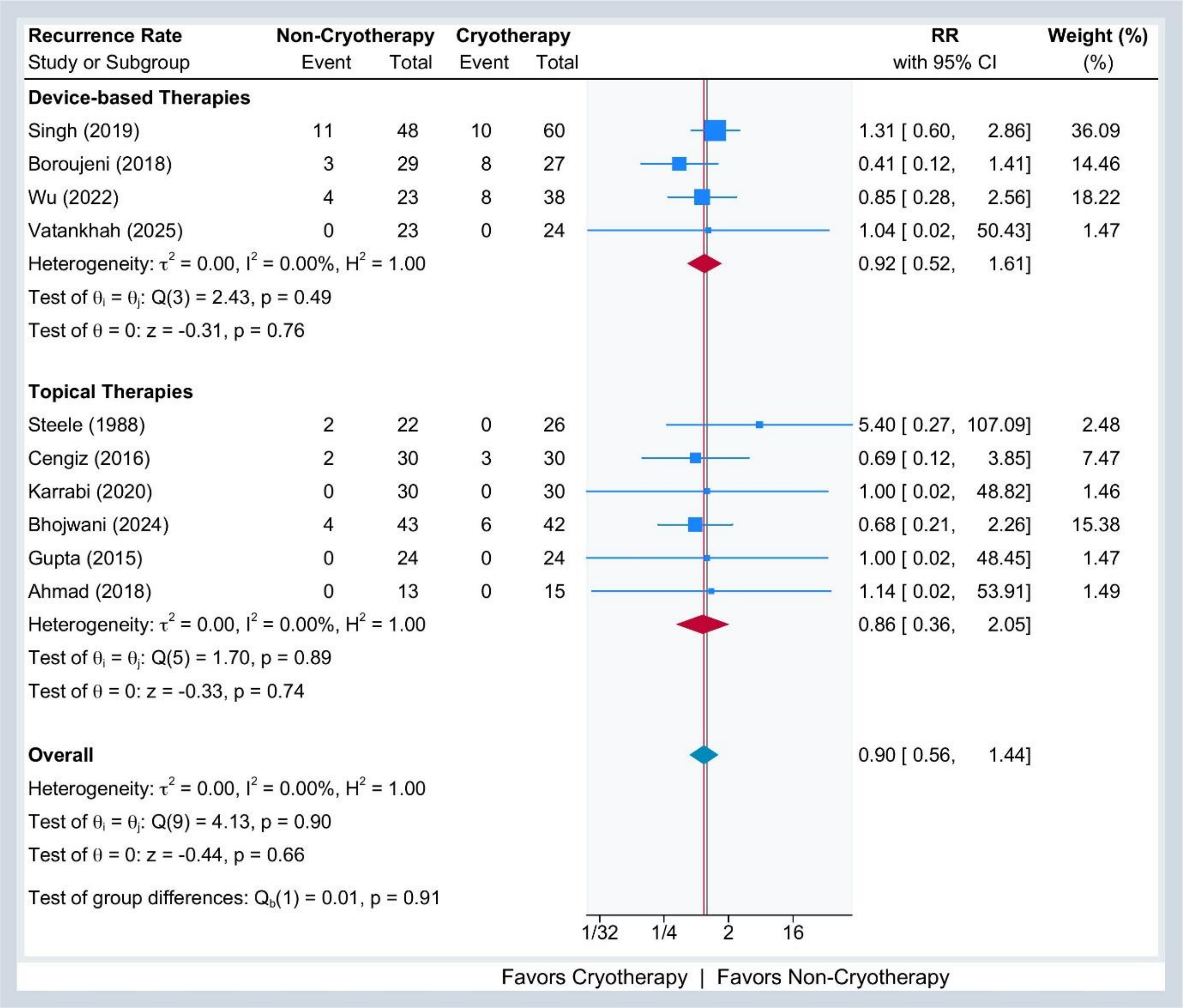

Forest plot comparing recurrence rates between non-cryotherapy interventions and cryotherapy in patients with plantar warts using a random-effects model.

RR = Risk Ratio; CI = Confidence Interval

Supplementary Figure S16. Forest plot of recurrence rate stratified by topical therapy mechanism of action.

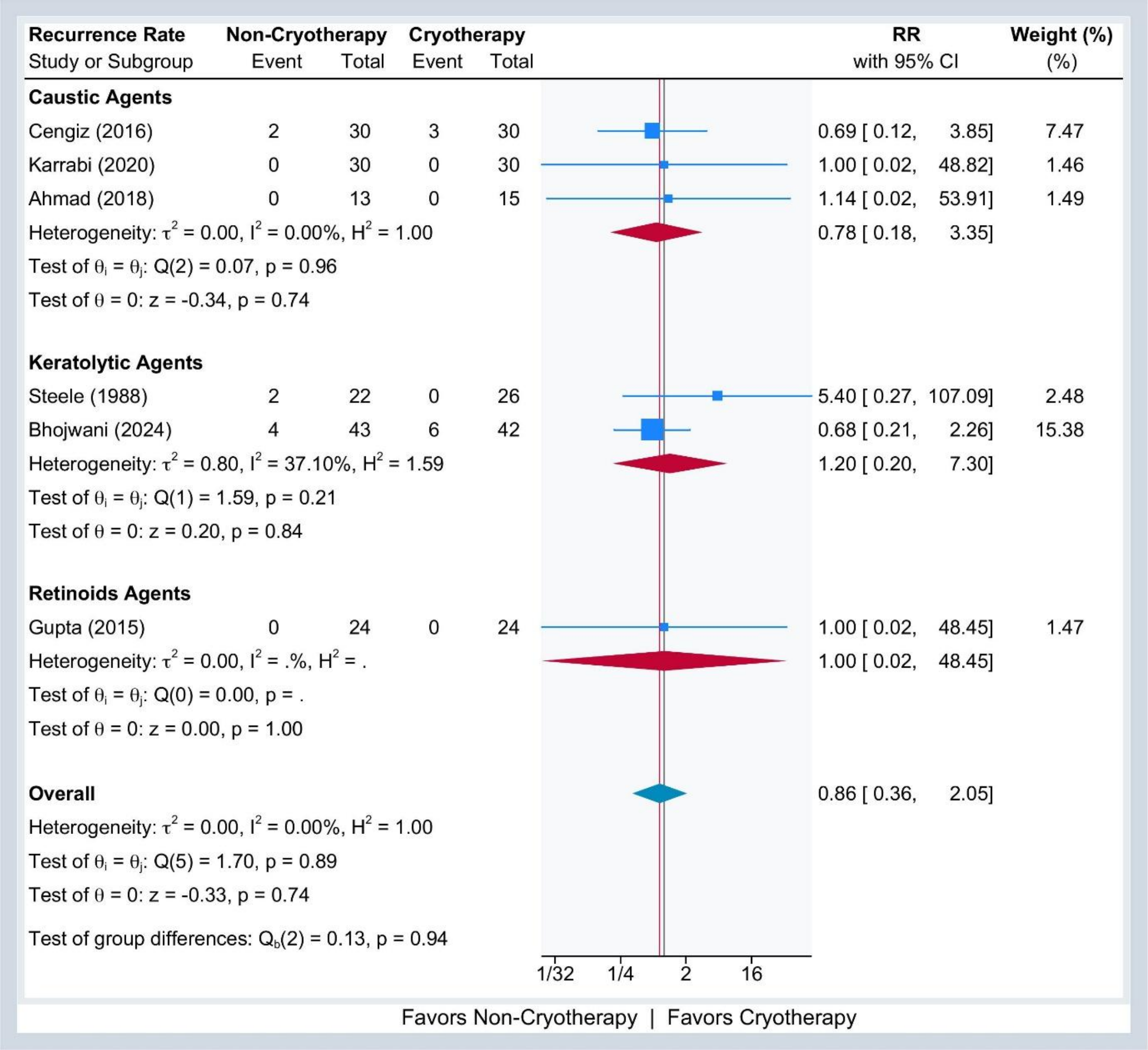

Forest plot comparing recurrence rates between topical non-cryotherapy interventions and cryotherapy, stratified by mechanism of action, using a random-effects model.

RR = Risk Ratio; CI = Confidence Interval

Supplementary Figure S17. Forest plot of recurrence rate stratified by cryotherapy application technique.

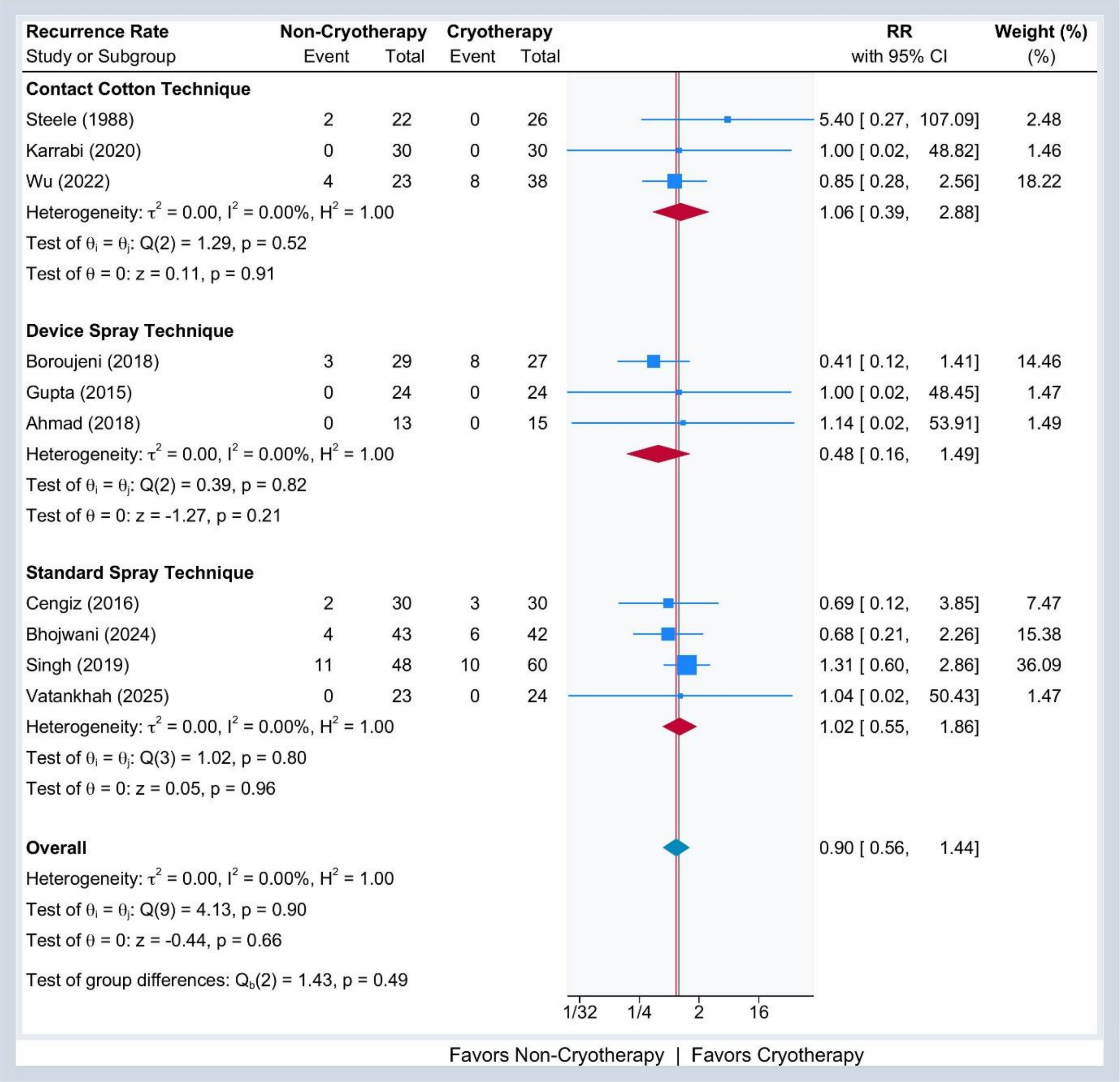

Forest plot comparing recurrence rates between non-cryotherapy interventions and cryotherapy, stratified by cryotherapy application technique, using a random-effects model.

RR = Risk Ratio; CI = Confidence Interval

Supplementary Figure S18. Forest plot of recurrence rate stratified by session frequency.

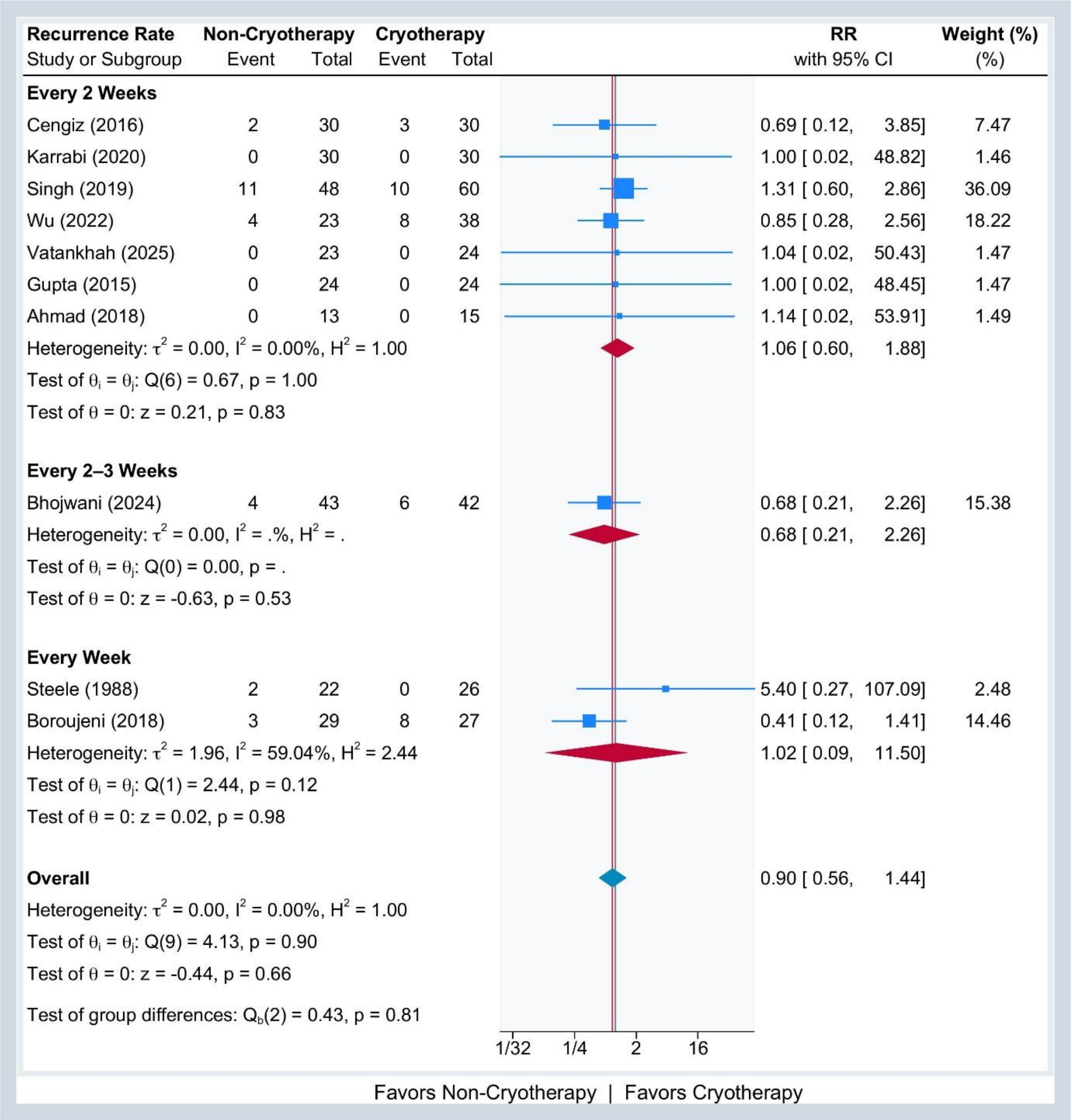

Forest plot comparing recurrence rates between non-cryotherapy interventions and cryotherapy, stratified according to cryotherapy session frequency, using a random-effects model.

RR = Risk Ratio; CI = Confidence Interval

Supplementary Figure S19. Forest plot of recurrence rate stratified by number of freeze–thaw cycles.

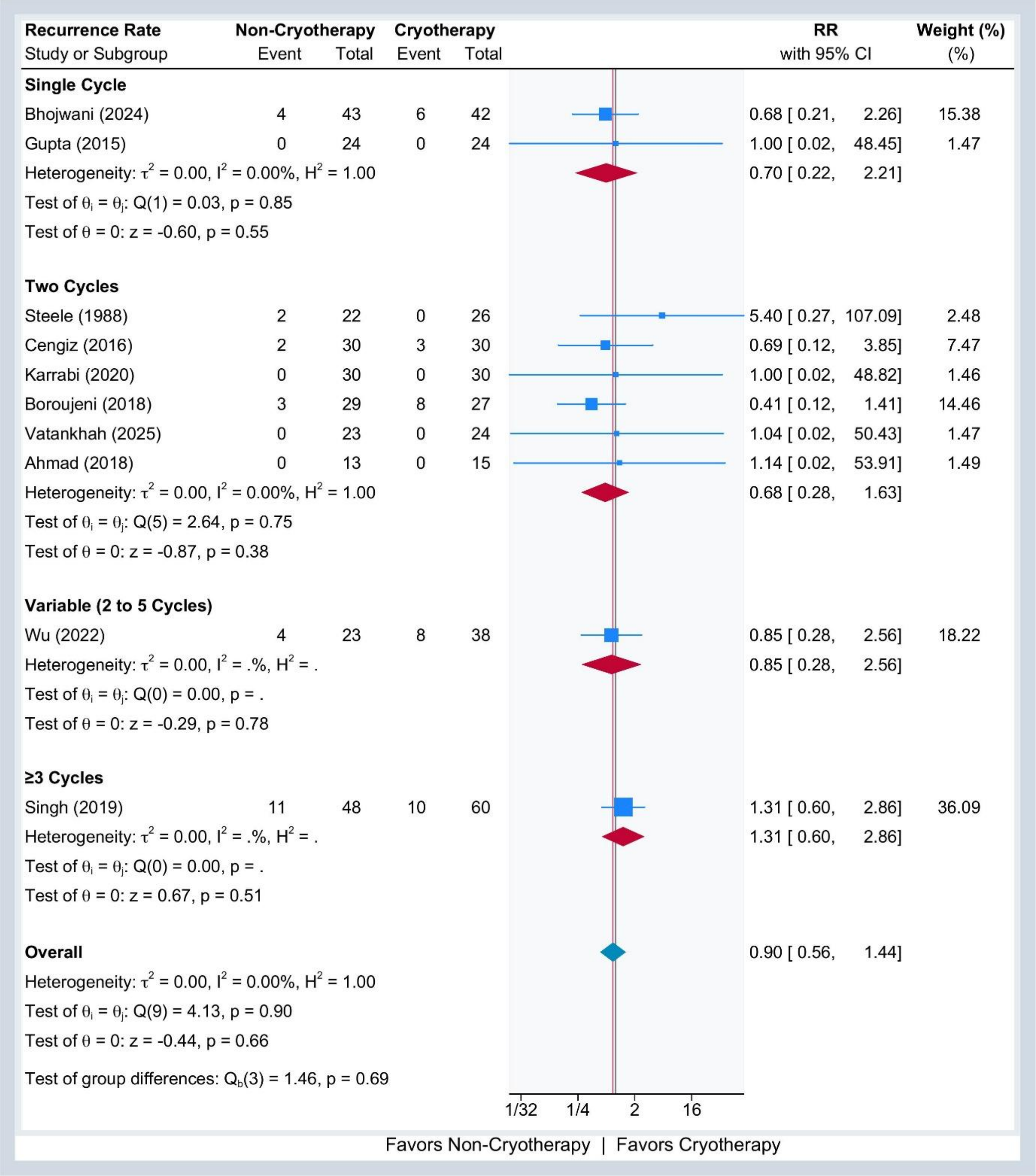

Forest plot comparing recurrence rates between non-cryotherapy interventions and cryotherapy, stratified according to the number of freeze–thaw cycles per session, using a random-effects model.

RR = Risk Ratio; CI = Confidence Interval

Supplementary Figure S20. Forest plot of recurrence rate stratified by freeze duration per cycle.

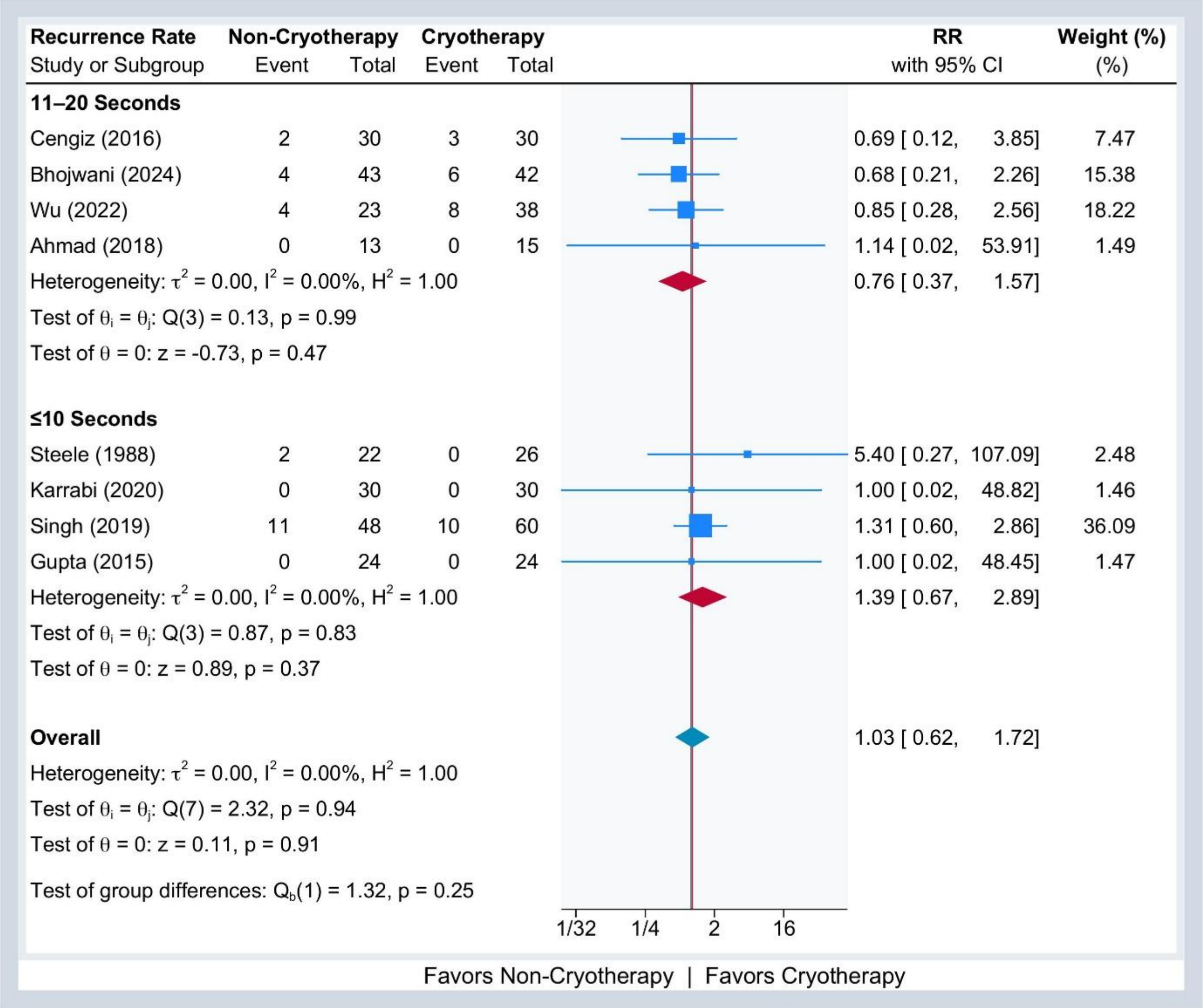

Forest plot comparing recurrence rates between non-cryotherapy interventions and cryotherapy, stratified according to freeze duration per cycle, using a random-effects model.

RR = Risk Ratio; CI = Confidence Interval

Supplementary Figure S21. Forest plot of blistering.

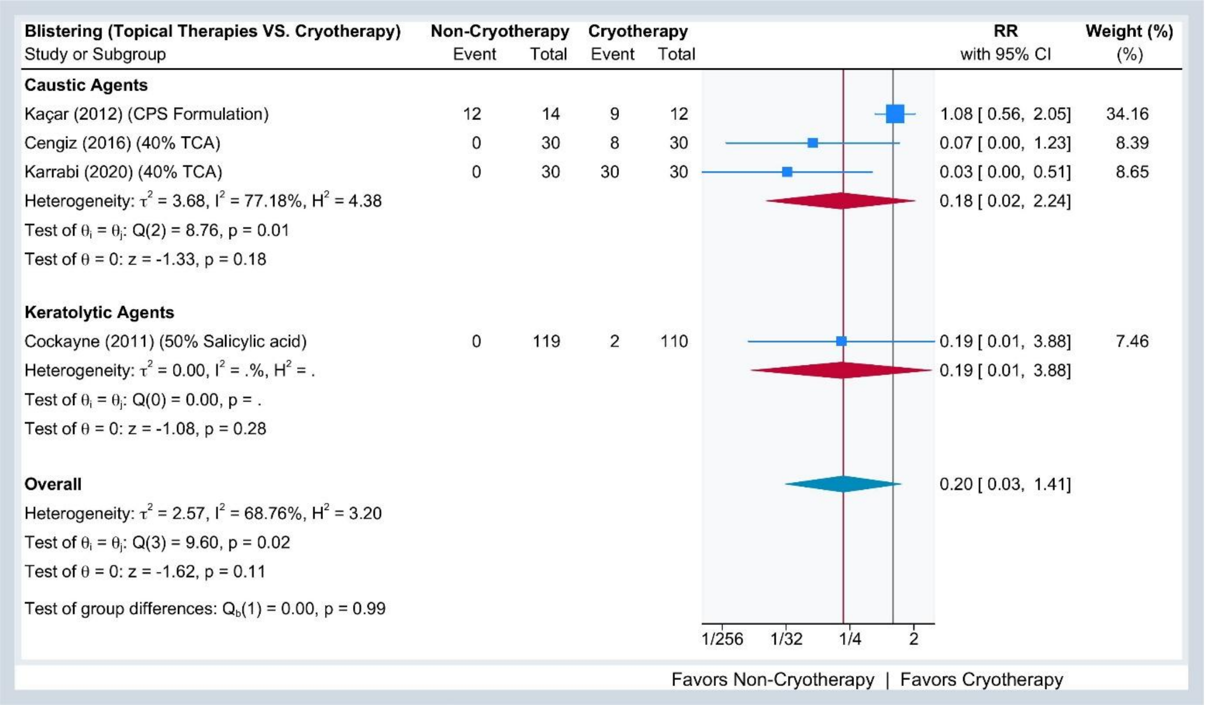

Forest plot comparing blistering rates between topical therapies and cryotherapy in patients with plantar warts using a random-effects model.

RR = Risk Ratio; CI = Confidence Interval

Supplementary Figure S22. Forest plot of scarring.

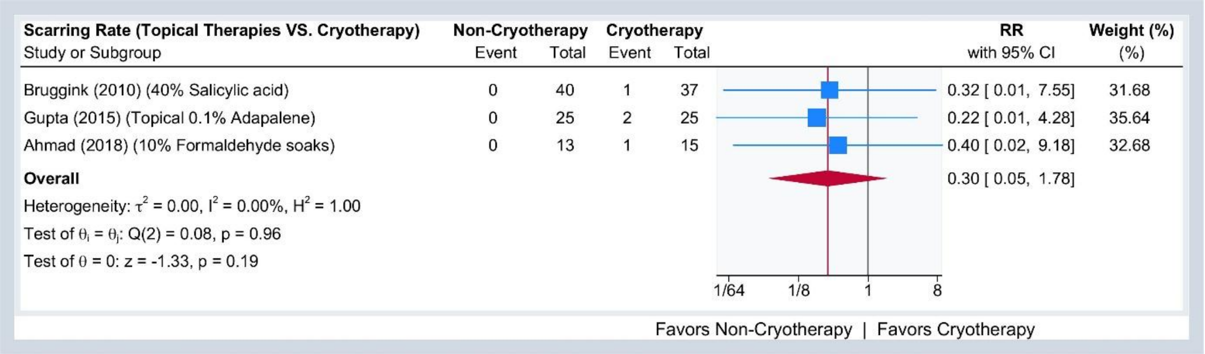

Forest plot comparing scarring rates between topical therapies and cryotherapy in patients with plantar warts using a random-effects model.

RR = Risk Ratio; CI = Confidence Interval

Supplementary Figure S23. Forest plot of secondary bacterial infection.

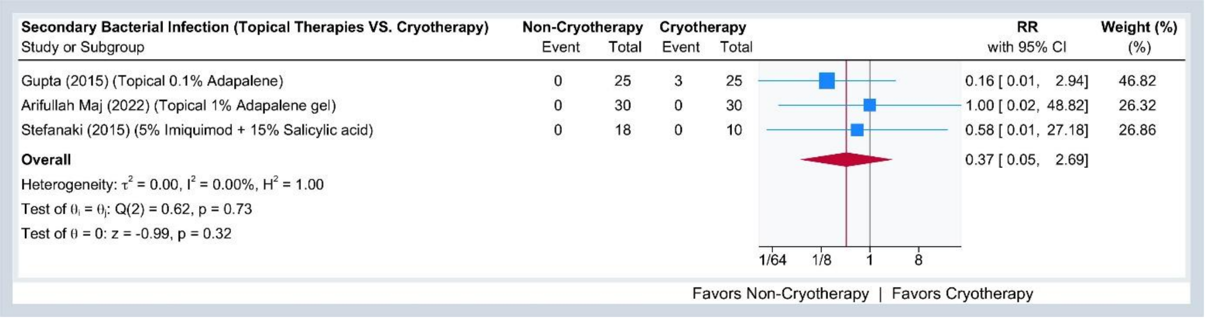

Forest plot comparing rates of secondary bacterial infection between topical therapies and cryotherapy in patients with plantar warts using a random-effects model.

RR = Risk Ratio; CI = Confidence Interval

Supplementary Figure S24. Forest plot of erythema.

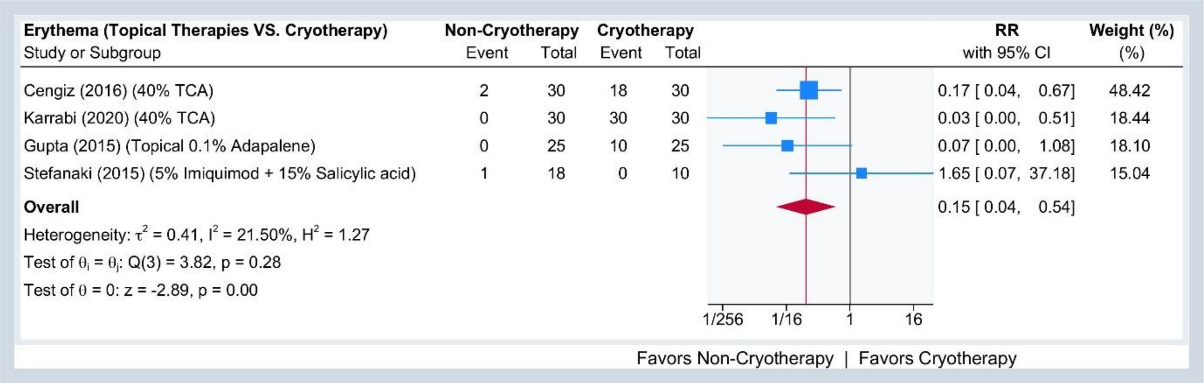

Forest plot comparing erythema rates between topical therapies and cryotherapy in patients with plantar warts using a random-effects model.

RR = Risk Ratio; CI = Confidence Interval

Supplementary Table S1. Detailed search strategy for each database.

Database	Search term	Filter	Results
**PubMed**	(Plantar Warts OR plantar wart* OR foot wart* OR feet wart* OR sole wart* OR verruca plantaris OR verrucae plantares OR human papillomavirus wart* OR myrmecia OR mosaic wart* OR recalcitrant plantar wart*) **AND** (cryotherapy OR cryosurgery OR cryoclearance OR intralesional cryotherapy OR intralesional cryosurgery OR freezing OR freeze-thaw OR freeze thaw cycle* OR liquid nitrogen OR LN2 OR cryospray OR cryo-spray OR cryogun OR cryo-gun OR cryogen OR cryoneedle OR cryoprobe OR cotton-tipped applicator) **AND** (salicylic acid OR trichloroacetic acid OR TCA OR calcipotriol OR monochloroacetic acid OR lactic acid OR formaldehyde OR nitric-zinc complex OR NZCS OR verrutop OR cantharidin OR podophyllin OR podophyllotoxin OR CPS OR bleomycin OR intralesional bleomycin OR 5-fluorouracil OR Chemotherapeutic OR Intralesional 5-FU OR 5-FU OR pingyangmycin OR imiquimod OR acyclovir OR intralesional acyclovir OR candida antigen OR PPD OR tuberculin OR MMR vaccine OR Intralesional Vitamin D3 OR vitamin D3 OR interferon OR transfer factor OR Carbon dioxide laser OR CO2 laser OR Erbium YAG OR Er:YAG OR pulsed dye laser OR Neodymium-doped Yttrium Aluminum Garnet OR Nd:YAG laser OR Photodynamic therapy OR photodynamic OR PDT OR Aminolevulinic acid OR 5-ALA OR duct tape OR silver duct tape OR duct tape occlusion OR microneedling OR radiofrequency ablation OR electrosurgery OR electrocautery OR electrodesiccation OR curettage OR surgical excision OR paring OR shaving OR adapalene OR retinoid OR zinc oxide OR tea tree oil OR placebo OR no treatment OR observation OR watchful waiting)	All Filed	**360**
**Scopus**	("Plantar Warts" OR "plantar wart" OR "foot wart" OR "feet wart" OR "sole wart" OR "verruca plantaris" OR "verrucae plantares" OR "human papillomavirus wart" OR "myrmecia" OR "mosaic wart" OR "recalcitrant plantar wart") **AND** ("cryotherapy" OR "cryosurgery" OR "intralesional cryotherapy" OR "intralesional cryosurgery" OR "freezing" OR "freeze-thaw" OR "freeze thaw cycle" OR "liquid nitrogen" OR "LN2" OR "cryospray" OR "cryo-spray" OR "cryogun" OR "cryogen" OR "cryoneedle" OR "cryoprobe" OR "cotton-tipped applicator") **AND** ("salicylic acid" OR "trichloroacetic acid" OR "TCA" OR "calcipotriol" OR "monochloroacetic acid" OR "lactic acid" OR "formaldehyde" OR "nitric-zinc complex" OR "NZCS" OR "verrutop" OR "cantharidin" OR	Article title, abstract, keywords	**280**

	"podophyllin" OR "podophyllotoxin" OR "CPS" OR "bleomycin" OR "intralesional bleomycin" OR "5-fluorouracil" OR "chemotherapeutic" OR "intralesional 5-FU" OR "5-FU" OR "pingyangmycin" OR "imiquimod" OR "acyclovir" OR "intralesional acyclovir" OR "candida antigen" OR "PPD" OR "tuberculin" OR "MMR vaccine" OR "intralesional vitamin D3" OR "vitamin D3" OR "interferon" OR "transfer factor" OR "carbon dioxide laser" OR "CO2 laser" OR "erbium YAG" OR "Er:YAG" OR "pulsed dye laser" OR "neodymium-doped yttrium aluminum garnet" OR "Nd:YAG laser" OR "photodynamic therapy" OR "photodynamic" OR "PDT" OR "aminolevulinic acid" OR "5-ALA" OR "duct tape" OR "silver duct tape" OR "duct tape occlusion" OR "microneedling" OR "radiofrequency ablation" OR "electrosurgery" OR "electrocautery" OR "electrodesiccation" OR "curettage" OR "surgical excision" OR "paring" OR "shaving" OR "adapalene" OR "retinoid" OR "zinc oxide" OR "tea tree oil" OR "placebo" OR "no treatment" OR "observation" OR "watchful waiting")		
**Web of Science**	(Plantar Warts OR plantar wart* OR foot wart* OR feet wart* OR sole wart* OR verruca plantaris OR verrucae plantares OR human papillomavirus wart* OR myrmecia OR mosaic wart* OR recalcitrant plantar wart*)<br>**AND**<br>(cryotherapy OR cryosurgery OR cryoclearance OR intralesional cryotherapy OR intralesional cryosurgery OR freezing OR freeze-thaw OR freeze thaw cycle* OR liquid nitrogen OR LN2 OR cryospray OR cryo-spray OR cryogun OR cryo-gun OR cryogen OR cryoneedle OR cryoprobe OR cotton-tipped applicator)<br>**AND**<br>(salicylic acid OR trichloroacetic acid OR calcipotriol OR monochloroacetic acid OR lactic acid OR formaldehyde OR nitric-zinc complex OR verrutop OR cantharidin OR podophyllin OR podophyllotoxin OR bleomycin OR intralesional bleomycin OR 5-fluorouracil OR Chemotherapeutic OR Intralesional 5-FU OR pingyangmycin OR imiquimod OR acyclovir OR intralesional acyclovir OR candida antigen OR PPD OR tuberculin OR MMR vaccine OR Intralesional Vitamin D3 OR vitamin D3 OR interferon OR transfer factor OR Carbon dioxide laser OR Erbium YAG OR pulsed dye laser OR Neodymium-doped Yttrium Aluminum Garnet OR Nd:YAG laser OR Photodynamic therapy OR photodynamic OR PDT OR Aminolevulinic acid OR duct tape OR silver duct tape OR duct tape occlusion OR microneedling OR radiofrequency ablation OR electrosurgery OR electrocautery OR electrodesiccation OR curettage OR surgical excision OR paring OR shaving OR adapalene OR retinoid OR zinc oxide OR tea tree oil OR placebo OR no treatment OR observation OR watchful waiting)	All Filed	**50**

**Cochrane library**	(Plantar Warts OR plantar wart* OR foot wart* OR feet wart* OR sole wart* OR verruca plantaris OR verrucae plantares OR human papillomavirus wart* OR myrmecia OR mosaic wart* OR recalcitrant plantar wart*)<br>**AND**<br>(cryotherapy OR cryosurgery OR cryoclearance OR intralesional cryotherapy OR intralesional cryosurgery OR freezing OR freeze-thaw OR freeze thaw cycle* OR liquid nitrogen OR LN2 OR cryospray OR cryo-spray OR cryogun OR cryo-gun OR cryogen OR cryoneedle OR cryoprobe OR cotton-tipped applicator)<br>**AND**<br>(salicylic acid OR trichloroacetic acid OR TCA OR calcipotriol OR monochloroacetic acid OR lactic acid OR formaldehyde OR nitric-zinc complex OR NZCS OR verrutop OR cantharidin OR podophyllin OR podophyllotoxin OR CPS OR bleomycin OR intralesional bleomycin OR 5-fluorouracil OR Chemotherapeutic OR Intralesional 5-FU OR 5-FU OR pingyangmycin OR imiquimod OR acyclovir OR intralesional acyclovir OR candida antigen OR PPD OR tuberculin OR MMR vaccine OR Intralesional Vitamin D3 OR vitamin D3 OR interferon OR transfer factor OR Carbon dioxide laser OR CO2 laser OR Erbium YAG OR Er:YAG OR pulsed dye laser OR Neodymium-doped Yttrium Aluminum Garnet OR Nd:YAG laser OR Photodynamic therapy OR photodynamic OR PDT OR Aminolevulinic acid OR 5-ALA OR duct tape OR silver duct tape OR duct tape occlusion OR microneedling OR radiofrequency ablation OR electrosurgery OR electrocautery OR electrodesiccation OR curettage OR surgical excision OR paring OR shaving OR adapalene OR retinoid OR zinc oxide OR tea tree oil OR placebo OR no treatment OR observation OR watchful waiting)	All Filed	**172**

Supplementary Table S2. Baseline characteristics of included studies.

StudyID	Groups	Sample size	Age (Years), Mean (SD) †	Male, n (%)	Number of warts, Mean (SD) †	Wart duration (months), Mean (SD) †
**Steele (1988) ***	SA/LA paint	22	(7-15) and ≥ 16 §	NR	NR	NR
	Cryotherapy	26		NR		NR
**Cockayne (2011)**	50% SA	123	30.2 (16.4)	50 (41)	3.4 (3.6) □	26.9 (24.5)
	Cryotherapy	117	30.1 (15.7)	31 (26)	4.0 (6.6) □	25.0 (24.9)
**Bruggink (2010) ***	40% SA	44	(4-12) and ≥ 12 §	30 (36)	2 (1–4) ¶	< 6 (37 (44)) ** ≥ 6 (47 (56))
	Cryotherapy	39		35 (44)	2 (1–3) ¶	< 6 (31 (39)) ** ≥ 6 (49 (61))
**Kaçar (2012)**	CPS	14	25.71 (8.08)	7 (50)	5.36 (4.03)	8.07 (9.20)
	Cryotherapy	12	23.92 (6.71)	5 (41.7)	4.91 (4.54)	18.50 (16.67)
**Cengiz (2016)**	40% TCA	30	20.03 (12.12)	13 (43.3)	NR	NR
	Cryotherapy	30	25.16 (9.04)	15 (50)	NR	NR
**Karrabi (2020)**	40% TCA	30	24.03 (12.11)	19 (63.3)	NR	35.5 (22.5)
	Cryotherapy	30	25.16 (8)	22 (73.3)	NR	37.8 (19.5)
**Bhojwani (2024)**	50% SA	43	34.6 (8.3)	24 (55.8)	2.1 (1.3)	6.8 (2.1)
	Cryotherapy	42	35.2 (7.9)	22 (52.38)	2.4 (1.4)	7.1 (2.3)
**García-Oreja (2023)**	NZCS	30	51.5 (17.7)	8 (26.7)	1.6 (1.1) □	1–6 (10 (33.3)) ** >12 (14 (46.7))
	Cryotherapy	32	50.3 (22.4)	13 (40.6)	2 (1.2) □	1–6 (10 (31.3)) ** >12 (171 (53.1))
**Singh (2020)**	Electrosurgery	71	25.2 (16-45) †*	71 (100)	4 (1-21) †*	29.3 (6-62) †*[a]
	Cryotherapy	76	25.7 (16-44) †*	76 (100)	4 (1-19) †*	27.4 (6-58) †*[a]
**Anwar (2021) ***	Electrocautery	15	22.72 (10.18)	15 (60)	NR	NR
	Cryotherapy	13	23.44 (13.45)	12 (48)	NR	NR
**Boroujeni (2018)**	CO_2 laser	29	27 (18-75) ‡	10 (34)	5 (1-20) ‡	NR
	Cryotherapy	27	25 (18-53) ‡	15 (56)	1 (1-20) ‡	NR
**Liu (2022) ***	LP-Nd:YAG Laser	55	28.5 (9.8)	51 (68)	NR	1.9 (2.3) [b]
	Cryotherapy	58	27.7 (8.2)	46 (61.3)	NR	1.5 (1.5) [b]
**Secolo (2025)**	Diode Laser	22	39 (16.72)	10 (45.45)	NR	NR
	Cryotherapy	22	36.77 (13.45)	10 (45.45)	NR	NR
**Wu (2022)**	ALA-PDT	23	27.36 (12.69)	12 (52.17)	NR	1.64 (0.85) [b]
	Cryotherapy	38	26.70 (7.45)	12 (31.57)	NR	1.79 (1.10) [b]
**Gibson (1984)**	Acyclovir cream	20	NR	NR	NR	NR
	Cryotherapy	12	NR	NR	NR	NR
**Azzazi (2026)**	Intralesional Acyclovir	34	33.35 (12.8)	26 (76.5)	51 ¤	36.68 ¶¶
	Cryotherapy	34	31.56 (13.145)	13 (38.2)	72 ¤	37 ¶¶
**Muhammad (2019)**	Intralesional Bleomycin	80	28.24 (4.2)	32 (40)	NR	1.25 (0.89)
	Cryotherapy	80	27.56 (3.8)	30 (36.5)	NR	0.98 (0.56)
**Vatankhah (2025) ***	Microneedling Bleomycin	23	34.40 (11.79)	14 (31.1)	2.04 (1.39) #	>1 year: 42 (93.3) †† >2 years: 2 (4.4) >3 years: 1 (2,2)
	Cryotherapy	24	32.91 (12.77)	19 (42.2)	1.70 (0.99) #	>1 year: 43 (95.6) †† >3 years: 2 (4.4)
**Khan (2023)**	Microneedling Mitomycin	30	20.13 (2.23)	22 (73.3)	3.50 (1.08)	NR
	Cryotherapy	30	25.31 (3.93)	21 (70)	3.47 (1.09)	NR
**Gupta (2015)**	0.1% Adapalene	24	28.58 (13-55) †*	11 (45.8)	286 ¤	NR
	Cryotherapy	24	25.7 (13-45) †*	14 (58.3)	124 ¤	NR
**Mahboob (2022)**	0.1% Adapalene	42	44.52 (8.13)	21 (50)	2.95 (1.68)	21.95 (5.96) [c]
	Cryotherapy	42	45.95 (8.28)	22 (52.38)	5.309 (2.46)	21.23 (4.98) [c]

**Abd El-Magiud (2020) ***	MMR vaccine	10	25.2 (6.03)	14 (70)	10.45 (8.49)	15.35 (15.03)
	Cryotherapy	10	28 (8.81)	12 (60)	7.75 (3.84)	9.6 (7)
**Shahzad (2019)**	Intralesional Vitamin D3	64	(18-35) §	38 (59.37)	1-3 §	1-12 §[a]
	Cryotherapy	64		31 (48.4)		
**Abdel-Latif (2020)**	Silver duct tape	50	30.75 (8.11)	46 (92)	2.34 (1.25)	6.11 (2.46)
	Cryotherapy	50	31.6 (10.33)	45 (90)	2.3 (1.44)	6.56 (2.27)
**Cunningham (2014)**	Needling	18	26.11 (9.99)	10 (56)	4.17 (3.68) □	28.61 (28.22)
	Cryotherapy	19	30.37 (12.65)	11 (58)	2.58 (2.71) □	45.74 (43.92)
**Usman (2024)**	50% SA	49	32.5 (10)	30 (61.2)	NR	NR
	Cryotherapy	49	30.2 (9.4)	30 (61.2)	NR	NR
**Chanal (2025)**	50% SA	41	38.4 (13.9)	10 (24.4)	41 ¤	12.9 (11.3)
	5% 5-FU	33	38.2 (13.3)	13 (39.4)	33 ¤	12.3 (9.1)
	5% Imiquimod	42	37.5 (15.6)	13 (31)	42 ¤	13.9 (19.8)
	Cryotherapy	36	40.9 (14)	7 (19.4)	36 ¤	20 (51.1)
**Ahmad (2018)**	10% Formaldehyde	13	18.4 (9.4)	10 (76.9)	6.38 (6)	4.7 (1.8)
	Cryotherapy	15	22.9 (12.9)	12 (80)	10.9 (16)	2.8 (1.8)
**El-Tonsy (1999)**	Nd:YAG laser hyperthermia	25	26.32 (10.59)	29 (58)	NR	5.48 (4.37)
	Cryotherapy	25			NR	
**Arifullah Maj (2022)**	1% Adapalene	30	32.2 (8.6)	17 (56.66)	NR	6.366 (2.95) [a]
	Cryotherapy	30	32.53 (8.77)	16 (53.33)	NR	6.5 (2.89) [a]
**Stefanaki (2015) ***	5% Imiquimod + 15% SA	18	8.06 ¶¶	51 (59.3)	NR	NR
	Cryotherapy	10			NR	NR

NR, Not Reported; **SA**, Salicylic Acid; **LA**, Lactic Acid; **SA/LA**, Salicylic Acid /Lactic Acid; **CPS**, Cantharidin-podophyllotoxin– salicylic acid; **TCA**, Trichloroacetic Acid; **NZCS**, Nitric-zinc complex solution; **CO_2**, Carbon Dioxide; **LP Nd:YAG**, Long-pulsed Neodymium-doped Yttrium Aluminum Garnet; **ALA**, Aminolevulinic acid; **PDT**, Photodynamic Therapy; **ALA-PDT**, Aminolevulinic acid Photodynamic Therapy; **MMR**, Measles, Mumps, and Rubella; **5-FU**, 5-Fluorouracil. *: Some included studies enrolled patients with mixed wart types. Baseline characteristics (e.g., age, sex, number of warts, and wart duration) were reported for the overall study population and were not stratified specifically for plantar warts except for the sample size. However, only plantar wart–specific outcome data were included in the quantitative synthesis. # Reported Plantar warts data only. † Continuous variables are presented as: Mean (SD), unless otherwise specified. § Reported as range. ‡ Reported as Median (range). ¶ Reported as Median (IQR). †* Reported as Average (range). ¤ Reported as the total number of warts. □ Number of warts was reported per patient rather than as a total for the study group. ** Duration of warts was reported as categorical data (number of warts, %). †† Duration of warts was reported as categorical data (number of patients, %). ¶¶ Mean only (SD not reported). [a] Duration reported in weeks. [b] Duration reported in years. [c] Duration reported in days.
